# ATP purinergic receptor signalling promotes Sca-1^+^ cell proliferation and migration for vascular remodelling

**DOI:** 10.1186/s12964-023-01185-2

**Published:** 2023-07-10

**Authors:** Yiqin Cui, Chunshu Li, Xinyi Zeng, Xiaoyu Wei, Pengyun Li, Jun Cheng, Qingbo Xu, Yan Yang

**Affiliations:** Key Lab of Medical Electrophysiology of Ministry of Education and Medical Electrophysiological Key Lab of Sichuan Province, Collaborative Innovation Center for Prevention and Treatment of Cardiovascular Disease, Institute of Cardiovascular Research, Southwest Medical University, Luzhou, China

**Keywords:** Sca-1^+^ stem cells, ATP, P2YRs, Migration, Proliferation, Vascular injury

## Abstract

**Aims:**

Vascular resident stem cells expressing stem cell antigen-1 (Sca-1^+^ cells) promote vascular regeneration and remodelling following injury through migration, proliferation and differentiation. The aim of this study was to examine the contributions of ATP signalling through purinergic receptor type 2 (P2R) isoforms in promoting Sca-1^+^ cell migration and proliferation after vascular injury and to elucidate the main downstream signalling pathways.

**Methods and results:**

ATP-evoked changes in isolated Sca-1^+^ cell migration were examined by transwell assays, proliferation by viable cell counting assays and intracellular Ca^2+^ signalling by fluorometry, while receptor subtype contributions and downstream signals were examined by pharmacological or genetic inhibition, immunofluorescence, Western blotting and quantitative RT-PCR. These mechanisms were further examined in mice harbouring TdTomato-labelled Sca-1^+^ cells with and without Sca-1^+^-targeted P2R knockout following femoral artery guidewire injury. Stimulation with ATP promoted cultured Sca-1^+^ cell migration, induced intracellular free calcium elevations primarily via P2Y_2_R stimulation and accelerated proliferation mainly via P2Y_6_R stimulation. Enhanced migration was inhibited by the ERK blocker PD98059 or P2Y_2_R-shRNA, while enhanced proliferation was inhibited by the P38 inhibitor SB203580. Femoral artery guidewire injury of the neointima increased the number of TdTomato-labelled Sca-1^+^ cells, neointimal area and the ratio of neointimal area to media area at 3 weeks post-injury, and all of these responses were reduced by P2Y_2_R knockdown.

**Conclusions:**

ATP induces Sca-1^+^ cell migration through the P2Y_2_R–Ca^2+^–ERK signalling pathway, and enhances proliferation through the P2Y_6_R–P38-MAPK signalling pathway. Both pathways are essential for vascular remodelling following injury.

Video Abstract

**Supplementary Information:**

The online version contains supplementary material available at 10.1186/s12964-023-01185-2.

## Introduction

Cardiovascular disease is the leading cause of death worldwide. Both acute cardiovascular events, such as myocardial infarction and stroke, and chronic events, such as atherosclerosis induce vascular remodelling, which may lead to reduced tissue blood supply and loss of cell function, resulting in organ failure and death [1]. Stem cells have the ability to migrate, self-renew and differentiate, and so can maintain tissue homeostasis and mediate regeneration and remodelling. Adult organs contain resident stem cells that remain static until they are activated by signals in the microenvironment associated with tissue damage or functional loss. Stem cell proliferation, migration and functional differentiation are highly coordinated processes involving multiple external ligands, receptors and intracellular signalling pathways. For example, stem cells differentiating into tissue adhesive cells often migrate while undergoing rear-to-front polarisation, protrusion, adhesion formation and rear retraction, termed the mesenchymal mode [2]. All of these steps are dependent on a large number of scaffold, adaptor and adhesion proteins, and each is regulated by distinct signalling pathways [3, 4]. Therefore, to develop effective interventions to regulate vascular remodelling, including small-molecule drugs and targeted stem cell therapies, it is essential to identify the molecular mechanisms regulating each step.

Resident stem/progenitor cells, which express markers such as Sca-1, c-Kit, CD34 and Flk-1, are present in all three layers of the artery. The migration, proliferation and differentiation of vascular progenitor cells expressing stem cell antigen-1 (Sca-1^+^ cells) in the adventitia contribute to angiogenesis, endothelial repair and neointima formation [5]. Here, we examined if these functions are mediated by purinergic receptor-induced calcium signalling. Cytoplasmic Ca^2+^ is a ubiquitous second messenger that transduces a variety of external physical and chemical stimuli into cellular responses, including cell migration and proliferation [6, 7]. Purinergic signals are the original form of intercellular communication in multicellular organisms. The release of purines such as adenosine and adenosine triphosphate (ATP) directly from damaged cells or from nearby mature cells can promote the migration and proliferation of surrounding cells. Once released, ATP triggers a series of signalling events [8], including elevation of cytoplasmic free calcium concentration ([Ca^2+^]_i_), through activation of purinergic ionotropic P2X receptors (P2XRs) and metabotropic P2Y receptors (P2YRs) on the cell surface. Activated P2XR channels are permeable to calcium [9], thereby allowing direct extracellular Ca^2+^ influx, while G-protein-coupled P2YRs activate phospholipase C (PLC), leading to the generation of IP_3_, activation of IP_3_ receptors on the endoplasmic reticulum (ER) and release of ER calcium stores. These two source of free Ca^2+^ then activate downstream signalling pathways and gene expression programmes required for migration and proliferation [10, 11]. Hypertension and atherosclerosis can damage vascular endothelial cells (ECs), causing the release of ATP, which acts on other ECs and smooth muscle cells through paracrine or autocrine transmission to promote proliferation and migration, ultimately leading to vascular remodelling [12].

In this study, we examined if stimulation of P2Rs is also involved in the migration and proliferation of Sca-1^+^ cells of the vascular wall. Based on our preliminary experiments, we hypothesised that extracellular ATP acts on Sca-1^+^ cell membrane P2YRs and coupled downstream signalling pathway to promote stem cell migration and proliferation. To provide direct evidence for these mechanisms, we first measured ATP-stimulated [Ca^2+^]_i_ by fluorometry, migration by transwell assays and proliferation by viable cell counting assays in isolated Sca-1^+^ cells, and then examined the effects of Sca-1^+^ cell-specific P2Y_2_R knockout in a mouse model of arterial injury. Collectively, these results confirmed our hypothesis that ATP-induced activation of P2Y_2_Rs and downstream calcium-dependent signalling pathways in Sca-1^+^ cells is essential for vascular remodelling after arterial injury.

## Methods

### Study approval

All animal experiments were conducted in accordance with the Guide for the Care and Use of Laboratory Animals published by the US National Institutes of Health and approved by the Ethics Committee of Southwest Medical University (protocol 201,903–190). Genetically modified animals (P2Y_2_R-Flox × Sca-1^+^-Cre mice and P2Y_2_R-Flox × Sca-1^+^-Cre × Rosa26-TdTomato mice) were purchased from the Shanghai Model Organisms Center (Shanghai, China, 913,100,007,030,557,379). Wild-type mice (WT, C57BL6/J background) were obtained from Chengdu Dossy Experimental Animals (Chengdu, China). All mice were anaesthetised through a nose cone (isoflurane 1.0% at a flow rate of 1.0 L/min) using a dedicated isoflurane evaporator, subjected to modelling surgery and then were euthanized by cardiac perfusion of phosphate-buffered saline under deep isoflurane anaesthesia (3.0% at a flow rate of 1.5 L/min) at the indicated time to collect tissue samples for further analysis.

### Isolation and culture of Sca-1^+^ cells

Sca-1^+^ cells were isolated from the thoracic aorta adventitia of C57Bl6/J mice as previously reported [13]. Briefly, primary cultured adventitia cells were screened using magnetic beads (Anti-Sca-1-VioBright FITC, Anti-FITC MicroBead) to obtain Sca-1 + cells. Purified Sca-1^+^ cells were cultured and used as suggested in the online Supplemental Methods 1.1.

### Transwell migration assays

Cells were seeded and cultured in an incubator at 37 °C under a humidified 5% CO_2_ atmosphere and treated according to on-line Supplemental Methods 1.2. In our experiment, we applied ATP to the top chamber of the multiwell in serum-free medium, while the bottom chamber was without ATP in complete medium. Cells that migrated through the well were counted under bright field microscopy at 100 × .

### Cell viability assay

Cells were seeded in the upper chambers of transwell plates at (2–3) × 10^5^ cells/mL while the lower chambers were filled with DMEM plus 10% FBS. Then cells were incubated and treated according to the methods in the online Supplemental Methods 1.3. After the Cell Proliferation and Cytotoxicity Assay Kit 8 (CCK8) reagent was added, the OD value at 450 nm was measured to estimate the number of viable cells.

### Intracellular calcium measurement

Intracellular free Ca^2+^ concentration ([Ca^2+^]_i_) was measured in cultured Sca-1^+^ cells using a TILLvisION 4.0 imaging system as described previously [14] and as described in the online Supplemental Methods1.4. Briefly, Sca-1 + cells were loaded with 5 μM fura-2/AM and [Ca^2+^]_i_ was estimated by the ratio of fluorescence emission according to the equation.

$$[\mathrm{Ca}2+]\mathrm{i }=\mathrm{ Kd }\times (\mathrm{Sf}2/\mathrm{Sb}2) \times (\mathrm{R }-\mathrm{ Rmin})/(\mathrm{Rmax }-\mathrm{ R})$$

where Kd is the dissociation constant for fura-2/calcium (224 nM), R is the ratio of the fluorescence emission evoked by 340- and 380-nm light excitation, Rmin is the minimum emission ratio in Ca^2+^-free Tyrode’s solution with 10-mM EGTA, Rmax is the maximum ratio in saturating [Ca^2+^] solution (10-mM [Ca^2+^] Tyrode’s solution) and Sf2/Sb2 is the ratio of fluorescence emission evoked by 380-nm excitation in Ca^2+^-free Tyrode’s solution and saturating [Ca^2+^] solution. Transient changes in [Ca^2+^]_i_ induced by ATP were measured and the effects of treatment factors were observed according to online Supplemental Methods 1.4.

### Mouse femoral artery guidewire injury model

Mouse femoral artery guidewire injury model were made according to the method in the online Supplemental Methods1.5. Briefly, deep and superficial branches of the femoral artery were identified and separated from the femoral vein at the distal end. The guidewire inserted into the artery and the guidewire reached the common iliac artery, pushed and pulled back the guidewire three times, and left for 3 min. After the guidewire was withdrawn, the artery was quickly ligated. The target segment of femoral artery was cut 3 weeks post-surgery and placed into a tube prefilled with OCT, frozen in liquid nitrogen and stored at -80 °C until analysis.

### Immunofluorescence

Sca-1^+^ cells treated with online Supplemental Methods 1.6. Images were obtained using a confocal microscope (Zeiss-LSM-980, Germany).

### Hematoxylin and eosin staining

Frozen tissue sections were prepared at 10-µm thickness and the sections were treated with online Supplemental Methods 1.7. Images were obtained using a Digital slice scanner (KF-PRO-002, China).

### RT-PCR

Total RNA was isolated from Sca-1^+^ cells and treated with online Supplemental Methods 1.8. The specific primer pairs used for estimation of gene expression levels are presented in Supplementary Table 1.

### Western blotting

Total membrane and phosphorylated proteins were extracted from Sca-1^+^ cells. Samples (30 μg) were separated by sodium dodecyl sulphate polyacrylamide gel electrophoresis (SDS-PAGE) and transferred to nitrocellulose (NC) membranes. Membranes were incubated with the indicated primary antibody and then with horseradish peroxidase (HRP)-conjugated secondary antibody (1: 2000). Antibodies included anti-P2Y2R (extracellular) (1:200, Alomone), anti-P2Y6R (extracellular) (1:400, Alomone), anti-caveolin-1 (D46G3 XP®Rabbit mAb, 1:1000, Cell Signalling Technology), phospho-p44/42MAPK(Erk1/2) (Thr202/Tyr204) (D13.14.4E) XP®RabbitmAb#4370 (1:2000, Cell Signalling Technology) and p44/42 MAPK (Erk1/2) (137F5) rabbit mAb (1:1000, Cell Signalling Technology). Refer to the online Supplemental Methods 1.9 for detailed methods.

### Transcriptomics

The effect of ATP on gene expression was examined using the Illumina sequencing platform as described in the online Supplemental Methods 1.10. The differentially expressed genes (DEGs) between groups were screened using DESeq2 according to criteria |log2(FoldChange)|> 1.0 and adjusted *P* (Padj) < 0.05. The threshold for functional enrichment was also Padj < 0.05. The results are publicly available at BioProject (https://www.ncbi.nlm.nih.gov/bioproject/) under accession number PRJNA900447.

### Statistical analyses

All data are expressed as mean ± standard deviation (SD). GraphPad Prism 8.4.2 (GraphPad Software) was used for all statistical analyses. The number of independent samples/animals in each group is indicated in the Results section. The normality of each dataset was first determined by Shapiro–Wilk test and Pearson omnibus tests. Normally distributed datasets of equal variance were compared by one-way analysis of variance or Student’s t-test as indicated, while datasets with inhomogeneity of variance were compared using the Mann–Whitney U-test. A *P* < 0.05 was considered statistically significant for all tests.

## Results

### H_2_O_2_ and hypoxia stimulate ATP release from Sca-1^+^ cells

Mechanical damage to vascular cells induces oxidative stress and hypoxia, leading to ATP release. Treatment with H_2_O_2_ can recapitulate oxidative stress (OS)-induced cell damage [15, 16], so to examine OS-evoked ATP release in vascular tissue, cultured human umbilical vein endothelial cells (HUVECs), vascular smooth muscle cells (VSMCs) and Sca-1^+^ cells were stimulated with different concentrations of H_2_O_2_ (10, 20, 40, 80 and 100 µM) for 12 h, and ATP concentration in the culture medium was measured. The concentration of ATP in the extracellular medium of HUVECs increased in parallel with H_2_O_2_ concentration (Fig. S1 A), peaking at 7.1-fold greater than the control group at 100 µM (*P* < 0.01, *n* = 3). The extracellular ATP concentration was also increased significantly in the culture medium of VSMCs and Sca-1^+^ cells by 100 µm H_2_O_2_ (1.4-fold vs. control, *P* < 0.01, *n* = 3 cultures; 1.2-fold *P* < 0.001, *n* = 3 cultures; Fig. S1B and C, respectively). We also examined ATP release in response to hypoxia and reoxygenation [17]. Again, ATP concentrations were higher after hypoxia compared to the control (normoxia) group (Fig. S1D, E and F, respectively), peaking at 1.6-fold of control in HUVEC cultures after 6 h (*P* < 0.001, *n* = 5), 8.4-fold in VSMC cultures after 9 h (*P* < 0.001, *n* = 3) and 1.6-fold in Sca-1^+^ cell cultures after 9 h (*P* < 0.01, *n* = 3). Thus, stimuli recapitulating vascular damage can induce ATP release from Sca-1^+^ cells.

### Expression of P2Rs on Sca-1 + cells and the effect of ATP on expression

To determine the P2R subtypes regulating migration and proliferation of Sca-1^+^ cells, we first measured P2XR and P2YR expression by PCR and found that Sca-1^+^ cells mainly expressed P2X_4_R, P2X_5_R, P2Y_2_R and P2Y_6_R mRNAs (Fig. S2A). Treatment with ATP had no effect on P2Y_2_R and P2Y_6_R mRNA expression levels as measured by RT-qPCR or on membrane protein levels as measured by Western blotting (Fig. S2A-E). Immunofluorescence staining confirmed P2Y_2_R and P2Y_6_R surface expression on Sca-1^+^ cells (Fig. S2F and G).

### ATP promotes Sca-1^+^ stem cell migration

**Fig. 1:**
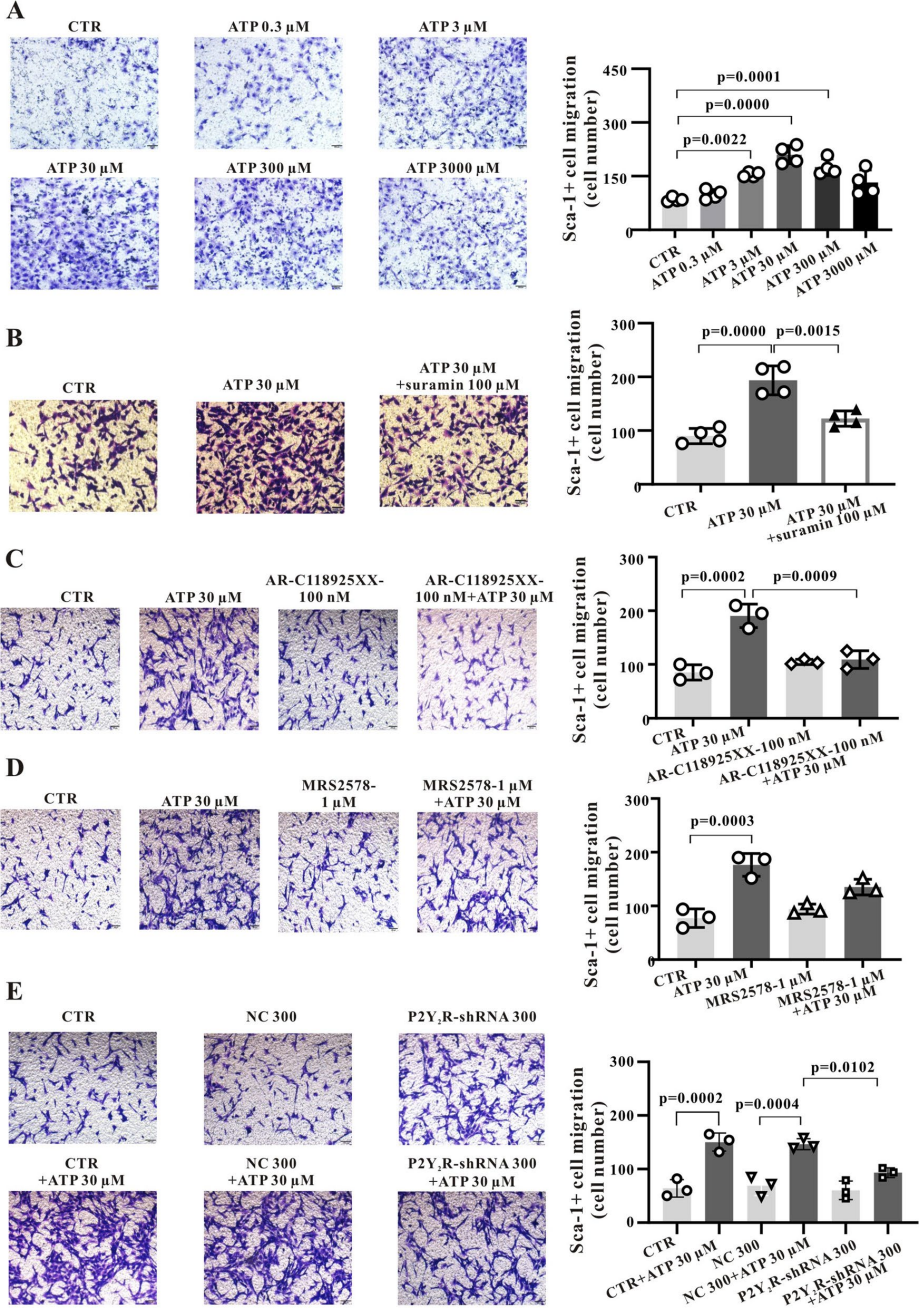
ATP promotes Sca-1^+^ stem cell migration through P2Y_2_R stimulation. A, Stimulation with ATP (0.3, 3, 30, 300 and 3000 µM) for 18 h dose-dependently promotes Sca-1^+^ cell migration in transwell assays. B, The non-specific P2R blocker suramin suppresses the facilitation of migration by ATP. C and D, The P2Y_2_R blocker AR-C118925 suppresses Sca-1^+^ cell migration induced by 30 μM ATP to a greater extent than the P2Y_6_R blocker MRS-2578. Images obtained at 100 × . E, A P2Y_2_R-shRNA also inhibits the migration of Sca-1^+^ cells induced by 30 μM ATP. Images obtained at 100 × . Summary data were obtained from 3–4 independent experiments. Data are expressed as mean ± standard deviation (SD). Data were first tested by Shapiro–Wilk test for normality. Ordinary one-way ANOVA (Tukey’s multiple comparisons test) was performed for A, C, D and E, while Brown-Forsythe and Welch One-way ANOVA (Dunnett’s multiple comparisons test) was performed for B. A *P* < 0.05 is considered a statistically significant test, and statistically significant P-values between the two groups are shown on the graph

Paracrine and autocrine ATP signalling pathways maintain tissue homeostasis, promote tissue repair and even contribute to disease pathogenesis by triggering migration and proliferation. To examine the influence of extracellular ATP on Sca-1^+^ cell migration, transwell assays were conducted. Treatment with 0.3–3000 µM ATP promoted migration of Sca-1^+^ cells (Fig. 1A, *P* < 0.01 to *P* < 0.001), with a peak effect at 30 µM. The non-specific P2R blocker suramin significantly inhibited ATP-stimulated transwell migration of Sca-1^+^ cells (Fig. 1B, *P* < 0.01).

Additional pharmacological and molecular methods were used to determine the main P2YR subtype(s) involved in Sca-1^+^ cell migration. The non-specific inhibitor suramin (100 μM for 5 min) reduced the number of migrating cells by about 80% (Fig. 1B, *P* < 0.01), while the P2Y_2_R specific blocker AR-C118925 (100 nM for 5 min) reduced the number of migrating cells by about 44% (Fig. 1C, *P* < 0.001). However, a high dose of the P2Y_6_R blocker MRS2578 [18] (1 μM for 10 min) reduced the number of migrating cells by only 14% (Fig. 1D). These results indicate that P2Y_2_R plays the predominant role and P2Y_6_R a secondary role in ATP-induced migration of Sca-1^+^ cells.

To provide additional evidence for the predominance of P2Y_2_R in promotion of Sca-1^+^ cell migration, a short hairpin (sh)RNA was designed to silence P2Y_2_R in Sca-1^+^ cells. Initial tests confirmed that this P2Y_2_R-shRNA could effectively downregulate P2Y_2_R expression, especially when the multiplicity of infection (MOI) was 300 or 500 (Fig. S3). At a MOI of 300, relative P2Y_2_R mRNA expression was reduced by 70% relative to control cells (Fig. S3A and B, *P* < 0.01), while P2Y_2_R protein expression was about 50% lower according to Western blot analysis (Fig. S1B and S3C and S3D, *P* < 0.01). Moreover, transfection with P2Y_2_R-shRNA reduced Sca-1^+^ cell migration induced by 30 µM ATP by about 23% compared to controls transfected with a scrambled shRNA (Fig. 1E).

### ATP promotes Sca-1^+^ stem cell proliferation

**Fig. 2:**
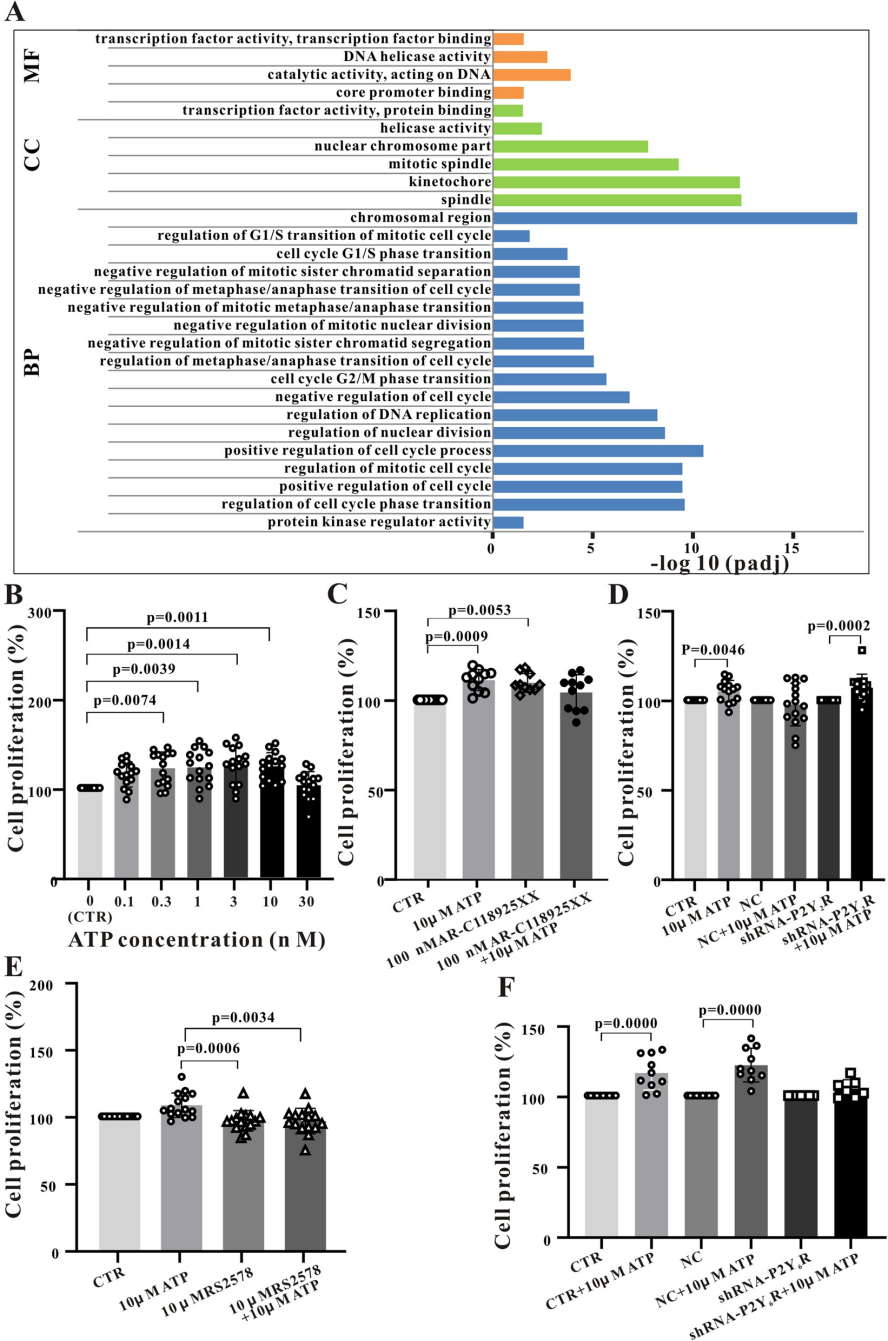
ATP promotes Sca-1 + cell proliferation through P2Y6R stimulation. **A**, Transcriptomic analysis revealing the main functional pathways involved in cell cycle changes induced by ATP. Molecular function (MF), cell component (CC) and biological process (BP). **B**, Effects of different ATP concentrations on the proliferation of Sca-1 + cells as measured by viable cell counting assay. **C**, The effects of ATP on proliferation are not markedly suppressed by the P2Y2R antagonist AR-C118925 (100 nM). **D**, Facilitation of proliferation by ATP is not blocked by specific P2Y2R knockout (D). **E** and **F**, Facilitation of proliferation by ATP is blocked by the P2Y6R antagonist MRS2578 (1 µM) (E) and by P2Y6R knockout (F). Summary data were obtained from 10–15 independent experiments. Data are expressed as mean ± standard deviation (SD). In B, C, and E figure, the indicated P-values were calculated using the Nonparametric test (Kruskal–Wallis test). In D and F figure, P-values were calculated using Mann–Whitney test and compared each group to the respective control. A *P* < 0.05 is considered a statistically significant test, and statistically significant P-values between the two groups are shown on the graph

Transcriptomic analysis revealed that ATP treatment altered the expression levels of genes involved in cell cycle control as indicated by functional pathway annotations ‘positive regulation of cell cycle’ and ‘chromosomal region’ (Fig. 2A). To confirm this effect, we conducted CCK8 assays and indeed found that treatment with 0.1–10 µM ATP for 16 h dose-dependently enhanced the rate of proliferation, and effect of 10 µM ATP treatment on Sca-1 + cell proliferation reached the peak, increasing to124.3% ± 3.923% (Fig. 2B, *P* < 0.001, *n* = 15 cultures).

To examine if P2Rs contribute to promotion of Sca-1^+^ cell proliferation by ATP, CCK8 experiments were repeated in the presence of suramin (100 µM). Compared to the control group, the cell proliferation rate increased to 112% ± 2.313% after 10 µM ATP treatment (*P* < 0.001, *n* = 11). After blocking P2Rs with 100 µM suramin, the proliferation of Sca-1 + stem cells promoted by 10 µM ATP was significantly inhibited, decreasing to 86.85% ± 3.841% (*P* < 0.001, *n* = 11) (Fig. S4). However, in contrast to migration, the P2Y_2_R-specific blocking agent AR-C118925 (100 nM) had no significant effect on proliferation (Fig. 2C). Similarly, knockdown of P2Y_2_Rs by shRNA adenovirus transfection had no effect on proliferation (Fig. 2D). In contrast, the P2Y_6_R-specific blocker MRS2578 (1 µM for 10 min) reduced proliferation. We found that 10 µM ATP increased Sca-1 + cell proliferation to 108.4% ± 2.38% (*P* < 0.01, *n* = 15). However, 1 µM MRS2578 could significantly inhibit this effect, and the proliferation was decreased to 96.55% ± 2.44% (*P* < 0.01, *n* = 15). (Fig. 2E). Similarly, P2Y_6_R knockdown by shRNA-P2Y_6_R adenovirus transfection inhibited proliferation in the presence of 10 µM ATP by 7.62% ± 4.11% (*P* < 0.05, *n* = 7) (Fig. 2F). Thus, P2Y_6_Rs make a greater contribution to ATP-induced acceleration of Sca-1^+^ cell proliferation than P2Y_2_Rs.

### ATP induces transient intracellular Ca^2+^ elevations in Sca-1^+^ cells

**Fig. 3:**
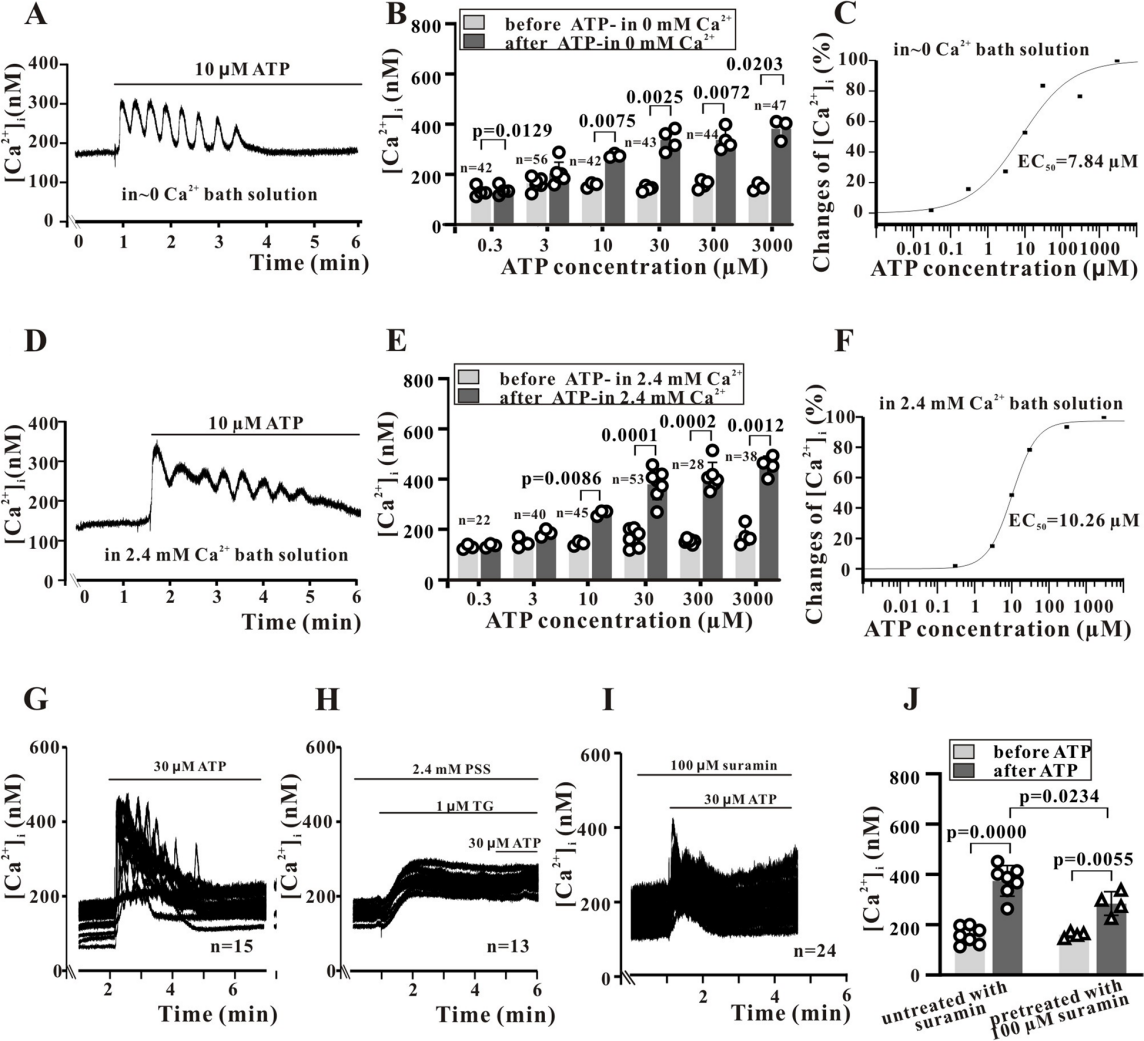
ATP-induced [Ca2 +]i oscillations in Sca-1 + cells. **A**, ATP-induced [Ca2 +]i oscillations in nominally 0 mM Ca2 + solution. **B**, Statistical histogram showing that ATP dose-dependently increases the peak [Ca2 +]i. **C**, ATP dose–response curve with Hill function showing an EC50 of 7.84 µM for ATP in nominally 0 mM Ca2 + solution. **D**-**F**, ATP-induced [Ca2 +]i elevations in 2.4 mM Ca2 + solution. G, Typical recordings showing that 30 µM ATP-induced robust [Ca2 +]i oscillations. **H**, Depletion of intracellular stores by 1 µM thapsigargin (TG) eliminates ATP-induced [Ca2 +]i oscillations. **I**, The non-specific P2Rs blocker suramin (100 µM) also inhibits ATP-induced [Ca2 +]i oscillations. **J**, Statistical histogram of suramin effects. Summary data were obtained from 3 to 7 independent experiments. The ‘n’ with number in the graph is the number of cells measured under each treatment condition. Data are expressed as mean ± standard deviation (SD). Data were first tested by Shapiro–Wilk test for normality and then paired t tests were performed for B and E, and Ordinary one-way ANOVA (Tukey’s multiple comparisons test) was performed for **J**. A *P* < 0.05 is considered a statistically significant test, and statistically significant P-values between the two groups are shown on the graph

As P2YRs are coupled to the PLC–inositol 1,4,5-triphosphate (IP3)–ER Ca^2+^ release pathway, we tested if ATP induces transient [Ca^2+^]_i_ elevations in Sca-1^+^ cells via ER release. Indeed, extracellular 10 µM ATP-induced [Ca^2+^]_i_ oscillations lasting about 3 min in the presence of nominally Ca^2+^-free Tyrode’s saline (Fig. 3A) and the peak of magnitude of these oscillations was dependent on ATP concentration (Fig. 3B), with an EC_50_ of 7.84 µM (Fig. 3C). These responses were similar in the presence of 2.4 mM extracellular Ca^2+^ (Fig. 3D, E and F), suggesting that intracellular Ca^2+^ release rather than Ca^2+^ influx is the main source of these [Ca^2+^]_i_ oscillations.

**Fig. 4:**
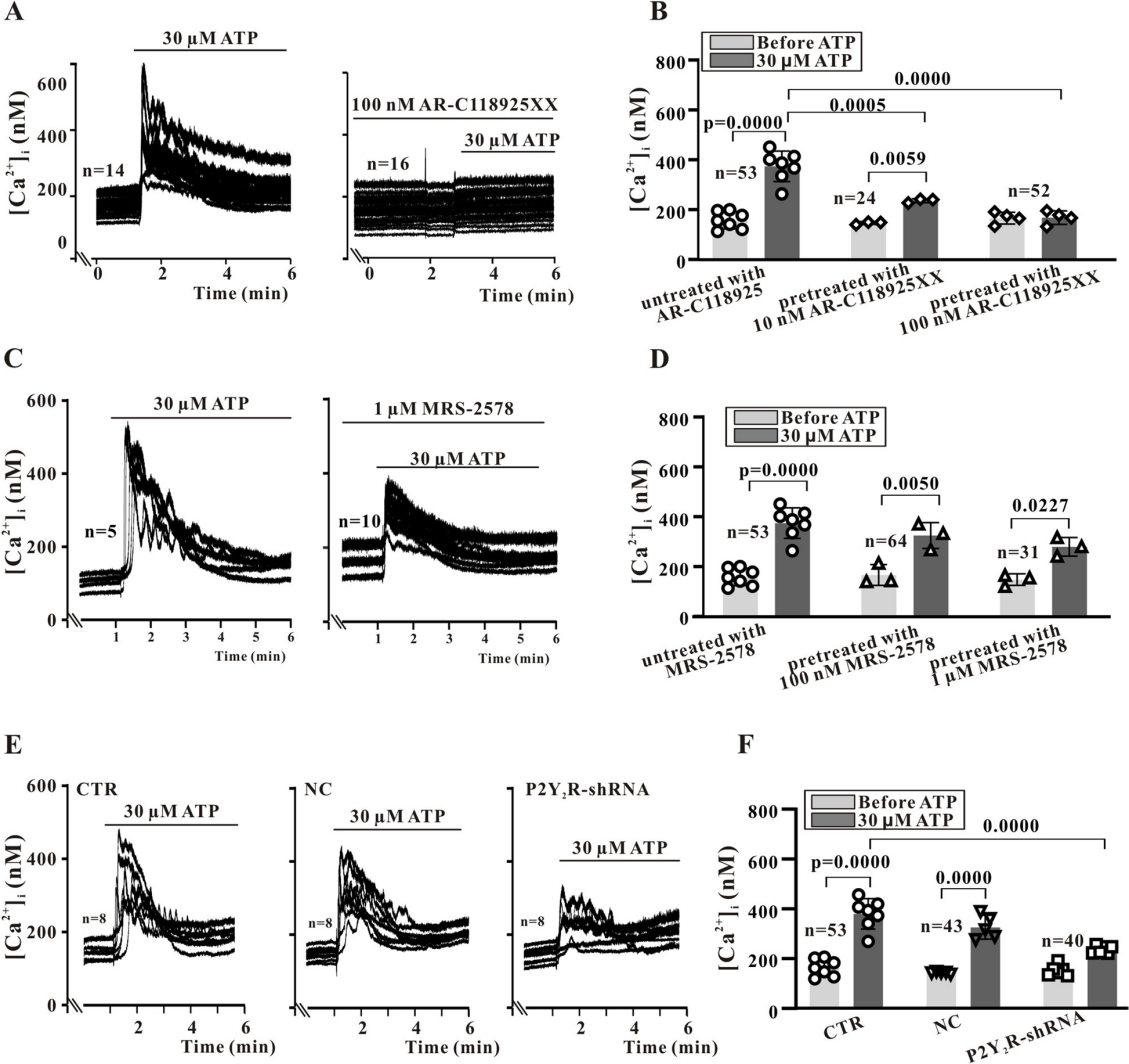
ATP-induced [Ca^2+^]_i_ elevations are mediated primarily by P2Y_2_Rs. **A** and **B**, The selective P2Y_2_R antagonist AR-C118925 significantly inhibits ATP-induced [Ca^2+^]_i_ elevations. **C** and **D**, The selective P2Y_6_R antagonist MRS-2578 has a weaker effect, even at a high concentration (1 μM). **E**, Typical records showing that control shRNA transfection does not significantly alter ATP-induced [Ca^2+^]_i_ elevations, while these [Ca^2+^]_i_ elevations are suppressed by P2Y_2_R-shRNA transfection. **F**. Statistical results showing that P2Y_2_R-shRNA significantly inhibits [Ca^2+^]_i_ elevations induced by 30 µM ATP. Summary data were obtained from 3 to 7 independent experiments. The ‘n’ with number in the graph is the number of cells measured under each treatment condition. Data are expressed as mean ± standard deviation (SD). Data were first tested by Shapiro–Wilk test for normality and then Ordinary one-way ANOVA (Tukey’s multiple comparisons test) was performed for B, D, and F. A *P* < 0.05 is considered a statistically significant test, and statistically significant P-values between the two groups are shown on the graph

To confirm ER release, we repeated these measurements following treatment with the ER calcium pump inhibitor thapsigargin (TG), which is known to deplete ER Ca^2+^ stores. Treatment with 1 µM TG nearly eliminated the [Ca^2+^]_i_ elevation induced by ATP (Fig. 3G vs. Figure 3H). Suramin (100 µM) also reduced the peak of [Ca^2+^]_i_ elevations induced by 30 µM ATP from 145.4% ± 24.3% (*n* = 53) to 81% ± 9.7% of control (*n* = 34 cells) (Fig. 3I and J). Similarly, the selective P2Y_2_R antagonist AR-C118925 (10 nM) inhibited [Ca^2+^]_i_ elevations in response to 30 µM ATP by approximately 55%, while 100 nM almost completely blocked the rise in [Ca^2+^]_i_ (Fig. 4A and B). However, even a very high concentration of MRS2578 (1 µM) inhibited peak [Ca^2+^]_i_ by only 25% (Fig. 4C and D), suggesting that the [Ca^2+^]_i_ changes in response to ATP are mediated primarily by activation of P2Y_2_Rs.

To confirm this function of P2Y_2_R, [Ca^2+^]_i_ measurements were repeated in Sca-1^+^ cells transfected with P2Y_2_R-shRNA (Fig. 4E and F). Transfection with a control shRNA reduced the response to 30 µM ATP from 145.9% ± 17.0% (*n* = 43) to 124.3% ± 8.2% (*n* = 27) (*P* < 0.05), while P2Y_2_R-shRNA reduced the [Ca^2+^]_i_ response from 145.9% ± 17.0% (*n* = 43) to 67.5% ± 8.9% (*n* = 41) (*P* < 0.001).

### Downstream signalling pathways involved in ATP-induced Sca-1^+^ cell migration and proliferation

To identify the downstream pathways influencing Sca-1^+^ cell migration and proliferation, we first conducted transcriptomic analysis of genes implicated in migration or proliferation and differentially expressed between control and ATP-treated cultures. A total of 54,533 genes were detected, of which 1763 were differentially expressed (1038 upregulated and 725 downregulated). Here, we focused on DEGs with migration-related and proliferation-related Gene Ontology (GO) terms.

Supplemental Fig. 5A (Fig. S5A) shows the main functional pathways involved in ATP-stimulated Sca-1^+^ cell migration according to GO analysis of DEGs, while the gene heat maps in Fig. S5B-E illustrate the involvement of specific DEGs in four representative functional pathways. This transcriptomic analysis suggests that ATP can influence migration of Sca-1^+^ cells through the Map2k1 gene, the Mapk3 gene, the MMP14 gene, the Rac1 gene and so on.

**Fig. 5:**
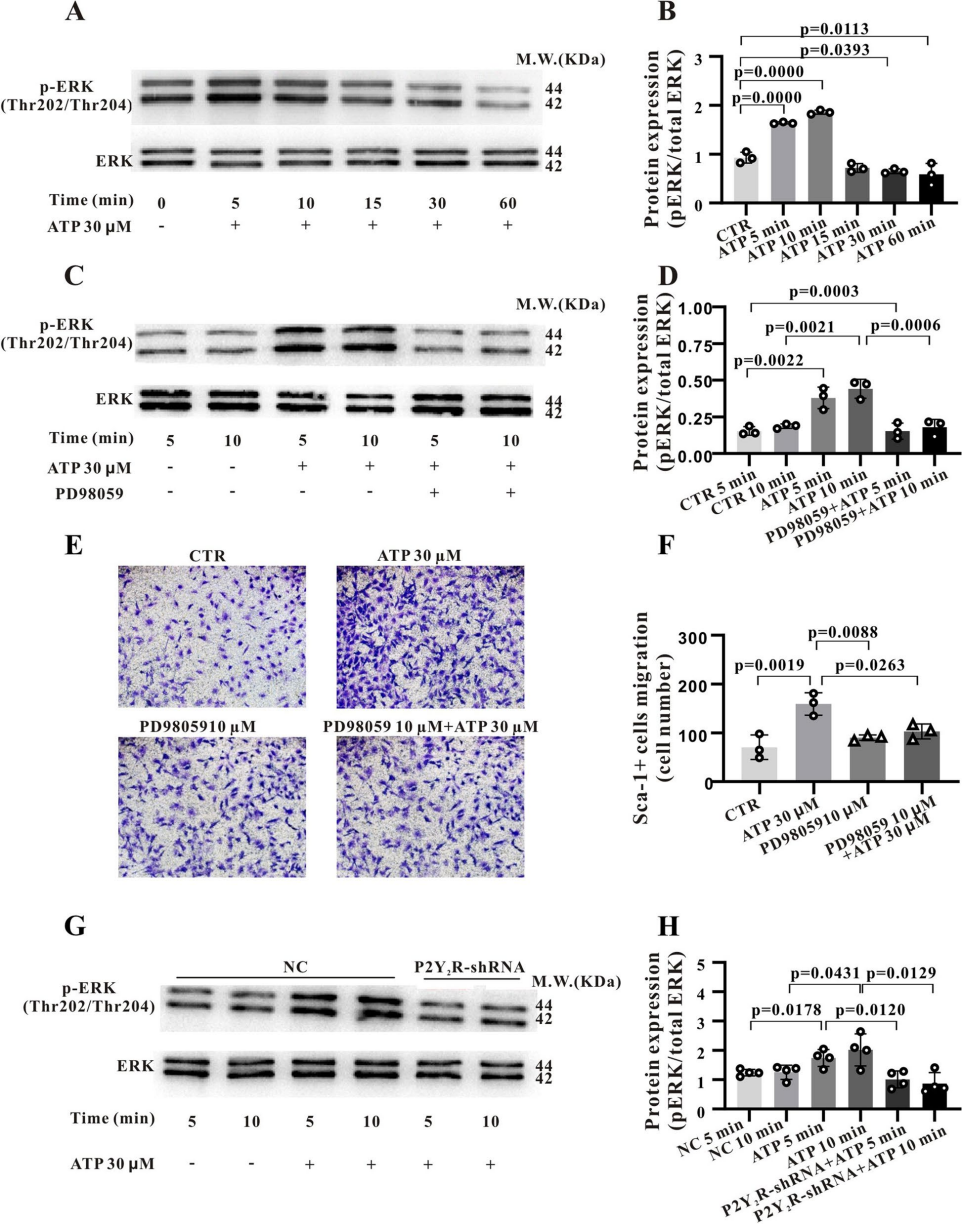
ATP-induced cell migration is mediated predominantly by the P2Y_2_R–ERK1/2 signalling pathway. **A** and **B**, Western blots showing that ATP (30 µM) activates ERK1/2 via Thr202/Tyr204 phosphorylation after 5 and 10 min of treatment. The histogram shows the relative activation level of ERK1/2 (pERK/ERK). **C** and **D**, ATP-activated ERK1/2 (Thr202/Tyr204 phosphorylation) is suppressed by the MEK inhibitor PD98059 (10 µM). **E** and **F**, ATP-activated migration is also suppressed by PD98059 (10 µM). **G** and **H**, P2Y_2_R-shRNA suppresses the phosphorylation of pERK1/2 induced by 30 μM ATP. Each statistic data in B, D, F were obtained from 3 independent experiments, while statistic data in H were obtained from 4 independent experiments. Data are expressed as mean ± standard deviation (SD). Data in B, D, F, and H were first tested by Shapiro–Wilk test for normality. Ordinary one-way ANOVA (Dunnett’s multiple comparisons test) was conducted for B, Ordinary one-way ANOVA (Tukey’s multiple comparisons test) was for F and unpaired t test was for D and H. A *P* < 0.05 is considered a statistically significant test, and statistically significant P-values between the two groups are shown on the graph

The extracellular signal-regulated kinase (ERK) pathway is activated by a variety of G protein-coupled receptors, and regulates the proliferation, differentiation and migration of many cell types. Moreover, strong [Ca^2+^]_i_ signals can activate ERK1/2 [19–21], so we examined potential ERK pathway activation in ATP-stimulated Sca-1^+^ cells and functions in migration. Stimulation with 30 μM ATP increased the phosphorylation (activation) of ERK1/2 (Fig. 5A and B, *P* < 0.001) as evidenced by Western blotting. Moreover, the ERK inhibitor PD98059 (10 μM) significantly reduced ERK1/2 phosphorylation after 5 and 10 min of 30 μM ATP stimulation (Fig. 5C and D, *P* < 0.001, respectively) and attenuated ATP-driven migration in transwell assays (Fig. 5E and F, *P* < 0.05). Similarly, transfection with P2Y_2_R-shRNA attenuated the phosphorylation of ERK1/2 induced by 30 μM ATP (Fig. 5G and H, *P* < 0.05). Collectively, these findings indicate that stimulation of P2Y_2_R by ATP induces activation of the ERK1/2 pathway, which in turn promotes the migration of Sca-1^+^ cells.

**Fig. 6:**
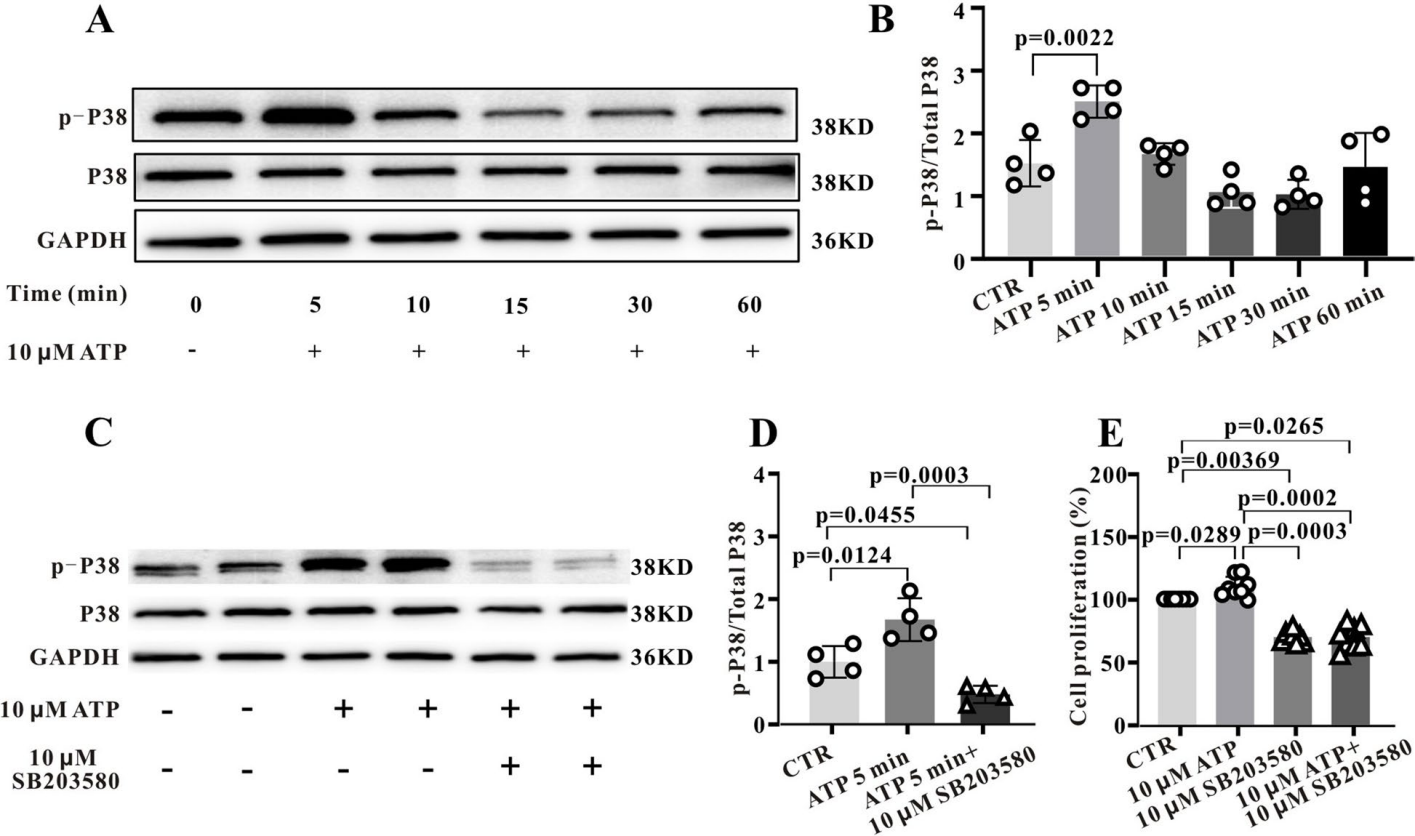
ATP-activated Sca-1^+^ cell proliferation is mediated predominantly by the p38-MAPK signalling pathway. **A**, Western blotting results showing enhanced p-P38 protein expression after 30-µM ATP stimulation for 5, 10, 15, 30 and 60 min. **B**, Statistical histogram of p-P38 protein expression levels after ATP stimulation for the indicated times. **C**, Western blot showing that P38 phosphorylation stimulated by ATP (30 µM, 5 min) is blocked by P38 inhibitor SB203580 (10 µM). **D**, Statistical histogram of p-P38 expression levels following cotreatment with ATP and 10-µM SB203580. **E**, SB203580 also blocks the promotion of Sca-1^+^ stem cell proliferation by ATP. Summary data were obtained from 4 independent experiments and data were first tested by Shapiro–Wilk test for normality and Ordinary one-way ANOVA (Dunnett’s multiple comparisons test) was performed for B and (Tukey’s multiple comparisons test) for D, while summary data were obtained from 8 independent experiments and the Nonparametric test (Kruskal–Wallis test) test was performed for E. A *P* < 0.05 is considered a statistically significant test, and statistically significant P-values between the two groups are shown on the graph

Mitogen-activated protein kinase (MAPK) signalling is one of the main pathways through which ATP affects proliferation as well as migration. Therefore, we conducted additional transcriptomic analysis of DEGs and found that cell cycle-related protein kinase activity was significantly altered by ATP treatment (Fig. S6A). Moreover, the overlap between genes upregulated or downregulated by ATP that influence proliferation in the transmembrane receptor protein tyrosine kinase signalling pathway (Fig. S6B) and in the positively regulate kinase activity pathway (Fig. S6C) suggest that MAPK-related genes contribute to the facilitation of Sca-1^+^ cell proliferation by ATP. To test this notion, we first measured changes in the phosphorylation (activation) status of MAPK isoform P38 in Sca-1^+^ cells stimulated by ATP (Fig. 6A), and indeed found that p-P38 expression was increased 1.7-fold after 5 min of ATP stimulation compared to control cells (*P* < 0.01, *n* = 4) ((Fig. 6B). Moreover, the P38 inhibitor SB203580 (10 µM) suppressed the ATP-induced increase in p-P38 by 68.88% ± 6.82% (*P* < 0.001, *n* = 4) ((Fig. 6C and D).

### ATP signalling and Sca-1^+^ cell responses contribute to regeneration and remodelling following femoral artery injury

**Fig. 7:**
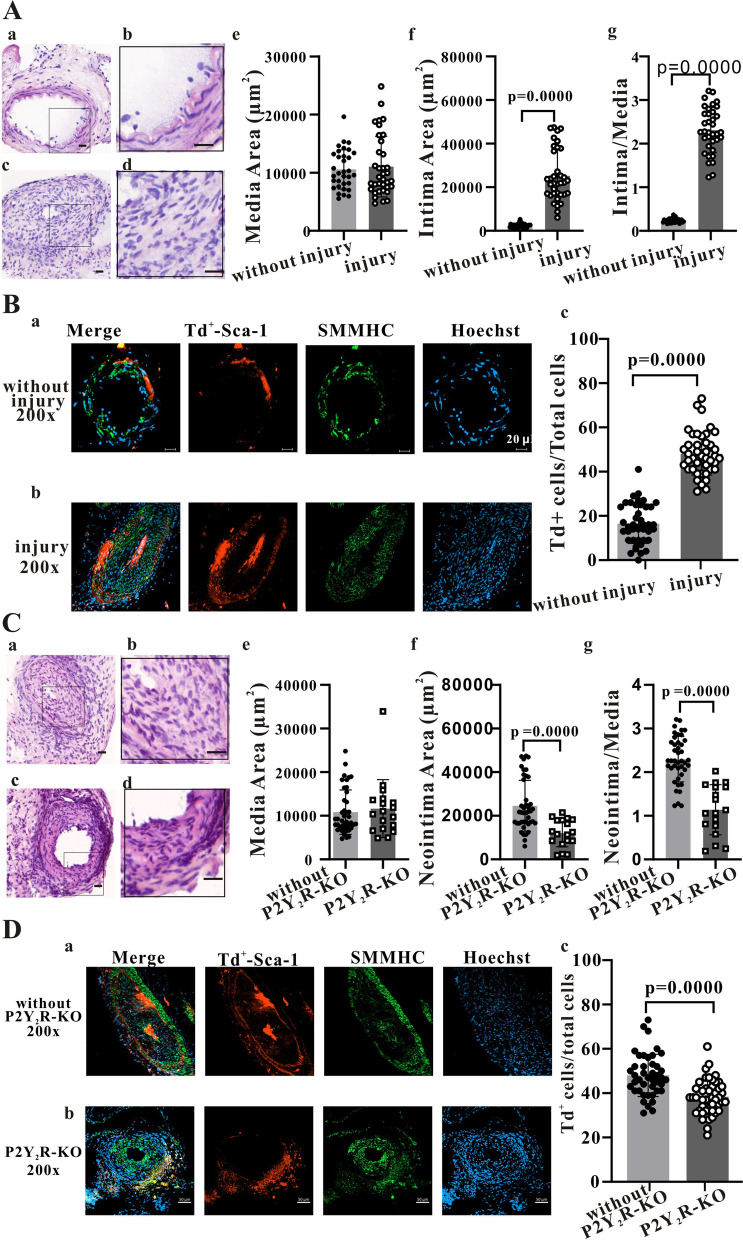
P2Y_2_R knockout suppresses remodelling of the mouse femoral artery following injury. **A**(a) and A(b), H&E staining showing a normal femoral artery. **A**(c) and A(d), H&E staining showing a thickened intima and obvious neointimal lesions 3 weeks after guidewire injury of the femoral artery. **A**(e)–A(g), Statistical summary showing the media area, neointima area and ratio of neointima area to media area. Summary data were obtained from 33 slices of 11 uninjured control mice and 36 slices of 12 injured mice. **B**(a)–B(c), Immunofluorescence images and statistical summary of femoral artery Sects. 3 weeks post-injury showing a significant increase in Sca-1^+^ cell number. Statistical summary data obtained from 42 samples of 9 uninjured mice and 43 samples of 9 injured mice. **C**(a)-C(g), H&E staining showing that P2Y_2_R knockout reduces the changes in neointima area and ratio of neointima area to media area following femoral artery injury. Summary data were obtained from 42 slices of 14 control mice and 18 slices of 6 P2Y_2_R knockout mice. **D**(a)–D(c), Immunofluorescence images and statistical summary showing that P2Y_2_R knockout partly reverses the rise in Sca-1^+^ cell/total cell ratio following injury. Summary data were obtained from 43 slices of 9 control mice and 44 slices of 6 P2Y_2_R knockout mice. Nonparametric test (Mann–Whitney test) was performed for all above statistical tests. A *P* < 0.05 is considered a statistically significant test, and statistically significant P-values between the two groups are shown on the graph. Red, Sca-1^+^ cells expressing TdTomato; Green, Markers of mature smooth muscles (SMHHC); Blue, Hoechst33342-stained nuclei. Scale bar in the H&E staining figures represents 20 µm

In this study, a 0.014-inch metal guidewire was used to establish a femoral artery injury model in C57BlJ/6 mice or C57BlJ/6 mice expressing TdTomato in Sca-1^+^ cells and lineages (Sca-1^+^-Cre × Rosa26-TdTomato mice). Hematoxylin and eosin staining three weeks post-injury revealed intimal hyperplasia, thickening and lesions (Fig. 7A*(a)*-*(g)*). Compared to the control femoral artery, intimal area was increased dramatically (*P* < 0.0001) from 2520 ± 992.7 µm^2^ (*n* = 33 slices from 11 uninjured control mice) to 25,202 ± 11,882 µm^2^ (*n* = 36 slices from 12 injury group mice) [Fig. 7A *(f)*], while the ratio of intimal area to medial area increased from 0.237 ± 0.048 to 2.311 ± 0.517 (*P* < 0.0001) [Fig. 7A *(g)*]. In addition, the proportion of Sca-1^+^ cells (indicated by red TdTomato fluorescence) among total cells increased significantly (*P* < 0.0001) from 16.45% ± 8.90% (*n* = 42 samples from 9 mice without injury) to 48.00% ± 9.57% (*n* = 43 samples from 9 mice in the injury group) (Fig. 7B).

Our in vitro results indicate that P2Y_2_R is the main receptor mediating the effects of ATP on migration and [Ca^2+^]_i_ in Sca-1^+^ cells. To confirm these functions of P2Y_2_R in the mouse model of vascular injury, we established a tamoxifen-inducible P2Y_2_R gene knockout mouse that still allows lineage tracing of Sca-1^+^ cells (P2Y_2_R-flox × Sca-1-CreERT × TdTomato, termed P2Y_2_R KO mice). Immunofluorescence confirmed that P2Y_2_R expression was markedly reduced in P2Y_2_R KO mice (Fig. S7), while H&E staining confirmed the extent of vascular injury in both the control mouse line Sca-1^+^-Cre × Rosa26-TdTomato and the P2Y_2_R KO line 3 weeks post-surgery [Fig. 7C *(a)–*7C*(d)*]. In contrast to the control line, however, the media area of P2Y_2_R KO mice did not change significantly post-injury, while the neointima area and the ratio of neointima area to media area were both significantly (*P* < 0.0001) lower in the P2Y_2_R KO group (12,111 ± 6305 µm^2^ vs. 24,572 ± 11,676 µm^2^ and 1.14 ± 0.57 vs. 2.30 ± 0.52; *n* = 42 samples from 14 control mice and *n* = 18 samples from 6 P2Y_2_R KO mice) [Fig. 7C*(e)–*7C*(g)*]. Also in contrast to control mice, P2Y_2_R mice exhibited significantly fewer (red fluorescent) Sca-1^+^ cells following injury (Fig. 7D). Further, the ratio of Sca-1^+^ cells to total cells was significantly lower in KO mice compared to control mice (38.41% ± 7.80% vs. 48.00% ± 9.57%, *n* = 44 samples from 6 KO mice and *n* = 43 samples from 9 control mice). These results suggest that specific knockdown of P2Y_2_R inhibits ATP-induced proliferation of Sca-1^+^ cells, thereby disrupting neointimal regeneration and remodelling following femoral artery injury. Finally, we examined the effect of P2Y_2_R KO on Sca-1 + cells after arterial injury by double immunostaining for smooth muscle cells, inflammatory cells, fibroblasts and endothelial cells. However, we found that P2Y_2_R KO was similar to control mice, except for endothelial cell staining. As shown in CD31images in Fig. S8, there was little co-stained Sca-1^+^ cells and ECs (red and green) in the KO group (inserted white arrow).

## Discussion

This study is the first to examine the functions of ATP in promoting the migration and proliferation of Sca-1^+^ cells during vascular remodelling. We found that ATP was released by cultured primary Sca-1^+^ cells and other vascular cells in response to hypoxic and oxidative stress conditions analogous to those detected during vascular injury, while direct ATP application dose-dependently induced [Ca^2+^]_i_ oscillations in Sca-1^+^ cells and accelerated both Sca-1^+^ cell migration and proliferation. Promotion of Sca-1^+^ cell migration was mainly mediated by P2Y_2_Rs and downstream activation of ERK1/2, while promotion of Sca-1^+^ cell proliferation was mainly mediated by P2Y_6_Rs and downstream activation of P38-MAPK. The number of labelled Sca-1^+^ cells was also significantly enhanced in mice 3 weeks following femoral artery guidewire injury, and this response was reduced by Sca-1^+^ cell-targeted P2Y_2_R KO. Collectively, these findings strongly suggest that ATP signalling in Sca-1^+^ cells contributes to regeneration and remodelling after femoral artery injury.

Under normal physiological conditions, cells contain millimolar concentrations of ATP in cytoplasm. Many cells types can also release vesicles containing ATP in response to mechanical stimulation, resulting in nanomolar to micro-molar concentrations within the extracellular space. For instance, it was reported that ATP is released in lung tissue during air inflation and that the release profile reflects the kinetics of stimulation [22], as prolonged (100 s) inflation triggered long-lasting ATP release that terminated only upon alveoli deflation/de-recruitment, while cyclic inflation/suction produced cyclic ATP release. Similarly, it was reported that oscillatory fluid flow induced the vesicular release of ATP from human mesenchymal stem cells [23]. In bone, ATP was rapidly released from osteoblasts in response to mechanical load and was dependent on Ca^2+^ entry through L-type voltage-sensitive Ca^2+^ channels [24]. ATP release is also be stimulated by injury. During inflammation, ischaemia, or hypoxia, damaged cells passively release ATP. For instance, damaged cells as well as local ECs, platelets and sympathetic nerves may release ATP during atherosclerosis, hypertension, restenosis and ischaemia, causing pathogenic vascular smooth muscle and endothelial cell proliferation. Our experiments support such release as cultured HUVECs, VSMCs and Sca-1^+^ cells released ATP in response to H_2_O_2_ and hypoxia. Numerous studies have also documented elevated ATP concentrations within the tumour microenvironment in vivo [25–28].

Sca-1^+^ cells can migrate and differentiate into a variety of cell types that participate in angiogenesis and remodelling [29]. Upregulation of leptin within the vascular walls and circulation after vascular injury has been reported to promote Sca-1^+^ cell migration and enhance neointima formation through a leptin receptor-dependent 3-Rac1/Cdc42-ERK1/2-pFAK pathway in db/db mice [13], while others have reported that Sca-1^+^ cells derived from embryonic stem cells can differentiate into ECs through HDAC3 activation, thereby accelerating the re-endothelialisation of injured arteries and reducing neointima formation. These Sca-1^+^ cell-derived ECs are a promising cell source for vascular engineering and blood vessel repair [29, 30]. Ingram et al. [31] also identified resident endothelial stem cells on the intima of umbilical veins. In addition, mesenchymal stem cells have been detected in human varicose saphenous veins that can differentiate into osteoblasts, chondrocytes and adipocytes in vitro [32]. Under physiological conditions, Sca-1^+^ cells are rare in the blood vessel wall, but numbers are significantly increased by injury. It is therefore of great clinical significance to explore the involvement of stem/progenitor cells and purinergic receptors in remodelling and intimal hyperplasia after injury. Here we showed that Sca-1 + cells residing on the vascular wall also contribute to neointima formation, whereas the downregulation of P2Y_2_R reduces neointima formation.

Increases in intracellular Ca^2+^ concentration can induce the expression of genes and the post-translational modification of proteins controlling migration, proliferation, apoptosis, motility, gene transcription and angiogenesis among other cellular processes. The specificity of the response is conferred by the spatiotemporal characteristics of the [Ca^2+^]_i_ increase, which in turn is determined by the subcellular localisation of intracellular Ca^2+^ release sites and Ca^2+^ influx pathways (e.g., voltage-gated ion channels) relative to calcium sequestration sites. Here we show that ATP elevates [Ca^2+^]_i_ by triggering Ca^2+^ release from the ER. It was also reported that ATP promoted the migration of human dental pulp mesenchymal stem cells (MSCs) and increased [Ca^2+^]_i_ [11, 33] and that P2Y_2_R triggered the migration of corneal epithelial cells after injury via extracellular Ca^2+^-dependent and Ca^2+^-independent pathways. Stimulation of P2Y_2_R was also found to activate the Src proto-oncogene, leading to the formation of Grb/Shc complexes and ERK signalling [8, 10]. It has been reported that ATP can stimulate MSC migration through P2X_7_R, P2Y_1_R and P2Y_11_R activation, and ensuing formation of a Ca^2+^ release-activated Ca^2+^ channel through the interaction of calcium release-activated calcium channel protein 1 (Orai1) and the matrix interaction molecule Sim1[11, 33]. Further, it was reported [34] that acetylcholine-induced MSC migration was reduced by the L-type and α1 calcium channel antagonist verapamil and by the Ca^2+^-ATPase inhibitor thapsigargin due to suppression of the Ca^2+^/PKC/ERK1/2 signal pathway. In the current study, extracellular ATP (0.3–3000 µM) dose-dependently increased [Ca^2+^]_i_, and we confirmed that both pharmacological inhibition and shRNA-mediated knockdown of P2Y_2_R significantly inhibited this ATP-induced [Ca^2+^]_i_ response. Our results demonstrate for the first time that Sca-1^+^ cell migration is promoted by [Ca^2+^]_i_ elevation mediated mainly by P2Y_2_R stimulation.

MAPKs are a group of serine-threonine kinases that transduce various exogenous stimuli into intracellular responses, including migration, proliferation, differentiation and apoptosis. When activated, MAPKs influence cellular metabolism, proliferation, morphology and survival through the phosphorylation of transcription factors, cytoskeletal proteins and other kinases. In mammals, there are three main MAPK signalling pathways: ERK1/2, JNK and P38 [35]. Our transcriptomic analysis suggested that MAPK-related genes implicated in migration and proliferation, such as MAPK3, Map2K1, Map2K3 and Map4K3, were significantly altered by ATP treatment, so mechanistic studies focused on the role of MAPK signalling in ATP-stimulated migration and proliferation. It is reported that the effects of extracellular nucleotides on proliferation and apoptosis depend on concentration and half-life in the extracellular medium, as well as the types of receptors activated. For example, nucleotides released by highly metastatic breast cancer cells activated P2Y_2_R, promoting tumour growth and invasion through crosstalk with ECs [36]. The migration of MCF-7 breast cancer cells was enhanced by activation of P2Y_2_R and downstream MEK-ERK1/2 signalling [37]. Similarly, the activation of Piezo1 channels stimulated the migration of human dental pulp-derived mesenchymal stem cells by inducing ATP release, subsequent P2R stimulation and activation of downstream PYK2 and MEK/ERK signalling pathways [38]. Other studies reported that ATP-stimulated hDP-MSCs mainly by activating P2X_7_Rs, P2Y_1_Rs, P2Y_11_Rs and Orai1/Sim1 channels. According to these aforementioned studies, it appears that MEK/ERK signalling molecules are frequently involved in the promotion of migration by ATP. We found that mouse vascular wall Sca-1^+^ cells expressed P2X_4_R, P2X_5_R, P2Y_2_R and P2Y_6_R mRNAs. Compared with the others, mRNA and membrane proteins of P2Y6R and P2Y2R were relatively strongly expressed. We also found that the ERK inhibitor PD98059 significantly inhibited ATP-induced cell migration and phosphorylation (activation) of ERK1/2. Further, P2Y_2_R knockout attenuated ATP-dependent phosphorylation of ERK1/2. These results highlight the importance of purinergic signalling in Sca-1^+^ cells and clarify the downstream signalling pathways controlling Sca-1^+^ cell migration.

In addition, ATP affects cell proliferation. In human dental pulp cells, low ATP concentrations promoted cell proliferation, while high concentrations inhibited proliferation [39]. ATP was also found to promote the proliferation of human glioma cells, and thus may substantially influence clinical progression. However, ATP inhibited the proliferation of adult human bone marrow MSCs. Our CCK-8 assays confirmed that 0.1–10 μM ATP promoted Sca-1^+^ cell proliferation and that this effect was mainly mediated by P2Y_6_R and downstream phosphorylation of P38-MAPK. It was reported that p-P38 protein level was increased during oleic acid-stimulated proliferation of VSMCs [40]. Moreover, studies have shown that ATP can activate P2X7R and P38 to induce T cell proliferation [41]. In the current study, p-P38 protein level was significantly elevated by ATP stimulation, while the P38 inhibitor SB203580 reduced the ATP-stimulated proliferation of Sca-1^+^ cells, suggesting that P38-MAPK signalling is the main downstream pathway controlling Sca-1^+^ cell proliferation following ATP stimulation of P2Y_6_Rs.

According to our transcriptome results, numerous other genes including those encoding Map2K, MMPs and Rac1 may regulate the ATP-induced migration or proliferation of stem cells. For example, PART1 promotes the proliferation, migration and invasion of hepatocellular carcinoma cells by regulating the miR-149-5p/Map2K1 axis [42], while NKILA induced by transforming growth factor βinhibited the expression of MMP14 and thereby suppressed the metastasis of oesophageal squamous cell carcinoma [43]. Moreover, the RhoG/Rac1 signalling pathway was found to promote the migration and invasion of salivary adenoid cystic carcinoma cells in part by activating Src/AKT/ERK1/2 signalling [44]. These results provide a foundation for studies aimed at identifying the genes and signal pathways controlling Sca-1^+^ cell migration and proliferation. The genes and proteins identified by our transcriptome results (Fig. S5 and S6) provide numerous targets for future research.

Several studies have proposed that Sca-1^+^ cells can differentiate into smooth muscle and ECs required for the regeneration of injured vessels [29]. In addition, recent lineage tracing studies have found that resident Sca-1^+^ cells in the vascular wall can differentiate into mature smooth muscle cells after injury [45]. Therefore, we constructed P2Y_2_R-flox × Sca-1-CreERT mice to observe the migration, proliferation and differentiation of Sca-1^+^ cells in the presence and absence of various MAPK and purinergic receptor antagonists. Guidewire injury resulted in both a greater neointima area and ratio of neointima area to media area in P2Y_2_R-expressing mice but decreased neointima area and ratio of neointima area to media area in P2Y_2_R knockout mice. The number of Sca-1^+^ cell was also increase by injury in P2Y_2_R-expressing mice but reduced in P2Y_2_R knockout mice following femoral artery injury, suggesting that ATP signalling through P2Y_2_Rs contributes to vascular regeneration and remodelling by promoting Sca-1^+^ cell proliferation and migration to the neointima. However, in the case of downregulation of P2Y_2_R in our study, there was little co-stained Sca-1 + cells and ECs. Hence, Sca-1^+^ might not contribute much to endothelial cell formation (Fig. S8).

Limitations of this study include the absence of P2Y_2_R or P2Y_6_R overexpression experiments and P2Y_6_R knockout experiments to distinguish the functions of these receptor subtypes on migration and proliferation in vivo. In addition, we did not examine the contributions of other signalling pathways. Additionally, the detection of ATP release at the site of injury in an animal model also reflects a limitation of this paper. The HEK293-PMELUC cells were created by stably transfecting HEK293 cells using a plasma membrane luciferin (pmeLUC) plasmid or virus. Our team also developed overexpressing HEK293 cells, called LV-OE-293 cells, which localized luciferase in the membrane. These cells were created to investigate any changes in ATP concentration near the injury site after femoral artery injury. Luminescence was detectable in both groups of mice at four days and three weeks post-surgery. However, we observed no significant enhancement in luminescence intensity at the injury site compared to the site without surgery (i.e., LV-OE-293 cells injected without surgery). One possible explanation for this observation is the timing of LV-OE-293 cell subcutaneous injection, which may have caused prompt inflammation, ATP release, and luminescence on both sides, thereby masking the luminescence from vascular injury-induced ATP release. To minimize bioluminescence caused by ATP release from mechanical, acute injury, or immediate inflammation, we attempted to inject the LV-OE-293 cells earlier (i.e., seven days before imaging) to expand the time interval between injection and imaging. However, due to the cell's lifetime limitation, we were unable to detect any luminescence and could not observe ATP release through these cells. Further research using more sensitive and effective methods is required to verify these findings.

## Conclusion

**Fig. 8:**
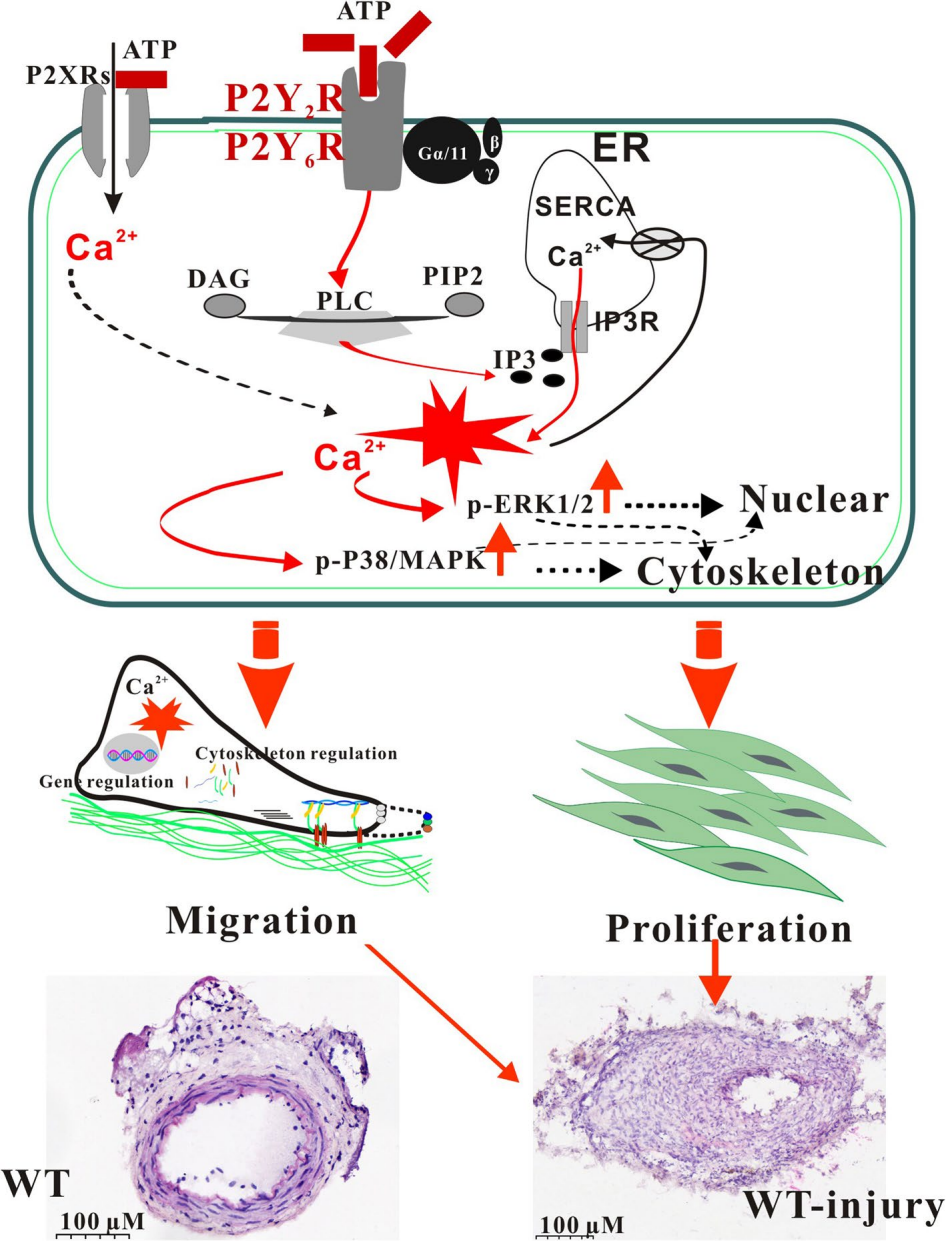
ATP purinergic signalling contributes to vascular remodelling after injury. ATP released from injured vascular cells binds to P2Y_2_Rs or P2Y_6_Rs on Sca-1^+^ cells, leading to a significant rise in intracellular Ca^2+^ concentration and activation of MAPK-ERK and MAPK-P38 signalling pathways. These MAPK pathways in turn regulate cytoskeletal dynamics and alter the expression levels of genes that promote Sca-1^+^ cell proliferation and migration. P2Y_2_R: purinergic metabotropic P2Y receptors type 2; P2Y_6_R: purinergic metabotropic P2Y receptor type 6; P2XRs: purinergic ionotropic P2X receptor channels; ER: endoplasmic reticulum; PLC: phospholipase C; p-ERK1/2: phosphorylated extracellular signal-regulated kinase 1/2; p-P38: phosphorylated P38 mitogen-activated protein kinase

Cardiovascular disease and vascular injury may lead to ATP release in blood vessel walls, where it acts as a stimulatory signal for Sca-1^+^ cell proliferation and migration. In this process, ATP binds to P2Y_2_Rs and P2Y_6_Rs, leading to the activation of MAPK-ERK and MAPK-P38 signalling pathways, respectively (schematic diagram shown in Fig. 8). Knockdown of P2Y_2_R decreased the neointima area and the ratio of neointima area to media area following vascular injury in mice, suggesting that ATP signalling via P2Y_2_Rs promotes neointimal formation in damaged vessels by triggering Sca-1^+^ cell proliferation. These findings enhance our understanding of the mechanisms underlying vascular diseases and identify promising targets for treatment.

## Supplementary Information

**Additional file 1: Materials and methods. Table 1. **Specific primer pairs for purinergic P2 receptor genes forand RT-PCR reagents. **Fig S1.** ATP release from Sca-1+ cells subjected to oxidative stress and hypoxia. **Fig S2.** mRNA and protein expression of P2R subtypes on Sca-1+ cell membranes. **Fig S3.** Testing for the optimal P2Y2R-shRNA transfection dose.** Fig S4.** Reduction in ATP-induced Sca-1+ cell proliferation by non-specific P2R blockade. **Fig S5.** Gene Ontologyterms related to cell migration or proliferation for genes differentially expressed between ATP-treated and control Sca-1+ cells. **Fig S6.** Gene Ontologyterms related to cell kinase activity for genes differentially expressed between ATP-treated and control Sca-1+ cells. **Fig S7.** Confirmation of P2Y2R knockdown in mouse femoral artery. **Fig S8.** Sca-1+ cell differentiation into smooth muscle cells, inflammatory cells, fibroblasts and endothelia cells. 

## Data Availability

The data supporting these findings are available from the corresponding author upon reasonable request.
