# Correction: ATP purinergic receptor signalling promotes Sca-1 ^+^ cell proliferation and migration for vascular remodelling

**DOI:** 10.1186/s12964-023-01222-0

**Published:** 2023-07-20

**Authors:** Yiqin Cui, Chunshu Li, Xinyi Zeng, Xiaoyu Wei, Pengyun Li, Jun Cheng, Qingbo Xu, Yan Yang

**Affiliations:** Key Lab of Medical Electrophysiology of Ministry of Education and Medical Electrophysiological Key Lab of Sichuan Province, Collaborative Innovation Center for Prevention and Treatment of Cardiovascular Disease, Institute of Cardiovascular Research, Southwest Medical University, Luzhou, China

**Correction: Cell Commun Signal 21, 173 (2023)**

**https://doi.org/10.1186/s12964-023-01185-2**

Following publication of the original article [1], the authors identified an error in affiliation 1. The correct affiliation is:

Key Lab of Medical Electrophysiology of Ministry of Education and Medical Electrophysiological Key Lab of Sichuan Province, Collaborative Innovation Center for Prevention and Treatment of Cardiovascular Disease, Institute of Cardiovascular Research, Southwest Medical University, Luzhou, China

The original article [1] has been corrected.
