# Supplementary material for: ATP purinergic receptor signalling promotes Sca-1+ cell proliferation and migration for vascular remodelling
Source: Cell Commun Signal. 2023 Jul 10;21:173. doi: 10.1186/s12964-023-01185-2 (PMC10332060; doi:10.1186/s12964-023-01185-2)
Supplement: Supplementary file 2 — Additional file 1: Materials and methods. Table 1. Specific primer pairs for purinergic P2 receptor genes forand RT-PCR reagents. Fig S1. ATP release from Sca-1+ cells subjected to oxidative stress and hypoxia. Fig S2. mRNA and protein expression of P2R subtypes on Sca-1+ cell membranes. Fig S3. Testing for the optimal P2Y2R-shRNA transfection dose. Fig S4. Reduction in ATP-induced Sca-1+ cell proliferation by non-specific P2R blockade. Fig S5. Gene Ontologyterms related to cell migration or proliferation for genes differentially expressed between ATP-treated and control Sca-1+ cells. Fig S6. Gene Ontologyterms related to cell kinase activity for genes differentially expressed between ATP-treated and control Sca-1+ cells. Fig S7. Confirmation of P2Y2R knockdown in mouse femoral artery. Fig S8. Sca-1+ cell differentiation into smooth muscle cells, inflammatory cells, fibroblasts and endothelia cells. [file 12964_2023_1185_MOESM1_ESM.pdf]

ATP purinergic receptor signalling promotes Sca-1+ cell proliferation and migration for vascular remodeling

Yiqin Cu†, Chunshu Li†, Xinyi Zeng, Xiaoyu Wei, Pengyun Li, Jun Cheng, Qingbo Xu*, Yan Yang*

Key Lab of Medical Electrophysiology of Ministry of Education and Medical Electrophysiological Key Lab of Sichuan Province, Collaborative Innovation Center for Prevention and Treatment of Cardiovascular Disease, Institute of Cardiovascular Research, Southwest Medical University, Luzhou, China

Correspondence to
Yan Yang, MD, email: wyangyan@swmu.edu.cn, or Qingbo Xu, PhD, email: qingbo_xu@zju.edu.cn, Key Laboratory of Medical Electrophysiology of Ministry of Education and Medical Electrophysiological Key Laboratory of Sichuan Province, Institute of Cardiovascular Research, Southwest Medical University, 1-1 Xianglin Road, Luzhou 646000, China Tel: +86 830-3161222.

Online Supplement

Files in this Additional file 1:

1. Materials and methods;
2. Supplementary table 1;
3. Supplementary Figures: Fig. S1, Fig. S2, Fig. S3, Fig. S4, Fig. S5, Fig. S6, Fig. S7, Fig. S8.

1. **Materials and methods**

1.1 Isolation and culture of Sca-1+ cells

Sca-1+ cells were isolated from the thoracic aorta adventitia of C57Bl6/J mice. Primary cultured adventitia cells were screened using magnetic beads (Anti-Sca-1-VioBright FITC, Anti-FITC MicroBead) to obtain Sca-1+ cells. Purified Sca-1+ cells were cultured in high-glucose American Type Culture Collection Dulbecco's Modified Eagle's Medium (ATCC DMEM) supplemented with 10% fetal bovine serum (FBS, Millipore, Burlington, MA, USA), 1% penicillin-streptomycin and 0.02% leukaemia inhibitory factor, and grown at 37°C in a humidified incubator under a 5% CO2 atmosphere. Cells were sorted with magnetic beads every 5 generations to maintain purity. All stem cells were used in experiments within 20 generations.

1.2 Transwell migration assays

Cells were seeded in the upper chambers of transwell plates at (2–3) × 105 cells/mL while the lower chambers were filled with DMEM plus 10% FBS. Cells were cultured in an incubator at 37°C under a humidified 5% CO2 atmosphere and treated as indicated (with ATP, the non-specific P2R blocker suramin, P2Y2R antagonist AR-C118925, P2Y6R antagonist MRS2578, adenovirus shRNA interference P2Y2R, adenovirus shRNA interference P2Y6R, and/or ERK1/2 inhibitor PD98059). Cells that migrated through the well were stained and counted under bright field microscopy at 100×.

1.3 Cell viability assay

Cells were seeded at 5 × 103 per well in 96-well plates with 100 µL complete medium per well and incubated overnight at 37°C under a humidified 5% CO2 atmosphere. After overnight adherent culture, the medium was exchanged for serum-free medium containing the indicated ATP concentration alone or ATP plus suramin (added 5 min in advance of ATP), ARC118925XX (added 5 min in advance), MRS2578 (added 10 min in advance), or SB203580 (added 1 h in advance). After ATP stimulation for 16 h, 10-µL CCK8 reagent was added to each well for 3 h. The OD value at 450 nm was measured to estimate the number of viable cells.

1.4 Intracellular calcium measurement

Intracellular free Ca2+ concentration ([Ca2+]i) was measured at room temperature (22°C–25°C) in cultured mouse Sca-1+ cells using a TILLvisION 4.0 imaging system (Till Photonics, Gräfelfing, Germany). Sca-1+ cells were loaded with 5 µM fura-2/AM for 30 min and [Ca2+]i was estimated by the ratio of fluorescence emission at 510 nm from 340 nm (15 ms) and 380 nm (10 ms) excitation according to the equation

$$[Ca2+]i = Kd \times (Sf2/Sb2) \times (R - Rmin)/(Rmax - R),$$

where Kd is the dissociation constant for fura-2/calcium (224 nM), R is the ratio of the fluorescence emission evoked by 340- and 380-nm light excitation, Rmin is the minimum emission ratio in Ca2+-free Tyrode's solution with 10-mM EGTA, Rmax is the maximum ratio in saturating [Ca2+] solution (10-mM [Ca2+] Tyrode's solution) and Sf2/Sb2 is the ratio of fluorescence emission evoked by 380-nm excitation in Ca2+-free Tyrode's solution and saturating [Ca2+] solution. The Tyrode's solution contained (mM) NaCl 127, KCl 5.9, CaCl2 2.4, MgCl2 1.2, glucose 12, HEPES 10 (pH adjusted to7.4 with NaOH) and was aerated continuously with 95% O2 and 5% CO2 during imaging. Nominally, 0 Ca2+ Tyrode solution contained no added CaCl2 and was supplemented with 0.5-mM EGTA. Transient changes in [Ca2+]i were evoked by applying ATP (0.3–3000 µM) in Tyrode's solution with 2.4-mM Ca2+ or in nominally 0 Ca2+ Tyrode solution. Cells were then treated with suramin, ARC118925, MRS2578, or P2Y2R shRNA as indicated and ATP-induced [Ca2+]i signals were measured again. The ATP dose–response curve was fitted with the Hill function
[increase % = Xn/(kn+Xn)],
where X is the ATP concentration and K is the Michaelis constant (EC50).

1.5 Mouse femoral artery guidewire injury model

Mice were anaesthetised using a dedicated isoflurane evaporator. Limbs were then fixed in the supine position, and the tongue was pulled out to prevent suffocation during the operation. The hair was removed from the lower limbs to expose the best surgical field of vision, the skin cut open with ophthalmic scissors along the femoral vein line and the subcutaneous tissue was exposed. The femoral vein, artery and nerve were observed from the inside out using a dissection microscope. The femoral nerve was separated by blunt dissection of the fascial tissue. Deep and superficial branches of the femoral artery were identified and separated from the femoral vein at the distal end. A hematoma was formed 1–2 cm distal from the deep–superficial branch point using a micro-tweezer clamp to facilitate entry of a syringe needle and guidewire. A 0.3 × 13 mm syringe needle was inserted at the hematoma site in the downward direction to prevent the tip from puncturing the contralateral vessel wall, and the needle was kept in position for 80 s to enlarge the rupture. The syringe needle was then withdrawn and the guidewire inserted immediately through the rupture. When the guidewire reached the common iliac

artery, it was pushed and pulled back three times, and left for 3 minutes to facilitate intimal injury. A 9-0 non-absorptive suture was placed below the femoral artery for later use. After the guidewire was withdrawn, the artery was quickly ligated, and about 0.4-cm of surgical suture was left in place to facilitate accurate sampling 3 weeks post-surgery. At that time, the target segment of femoral artery was cut and placed into a centrifuge tube prefilled with OCT, frozen in liquid nitrogen and stored at -80°C until analysis.

1.6 Immunofluorescence

Sca-1+ cells treated as indicated on sterile glass slides were washed lightly with PBS, fixed with 4% paraformaldehyde (PFA), blocked with BSA and incubated overnight with anti-P2Y2 receptor (extracellular) antibody (1:200, Alomone, Jerusalem, Israel, #APR-102) and anti-P2Y6 receptor (extracellular) Antibody (1:1000, Alomone, #APR-106). The next day, cells were treated with secondary antibody (donkey anti-rabbit IgG (H+L) Highly Cross-Adsorbed or conjugated to Alexa Fluor 488 (1:500, Thermo Fisher). HelixGen anti-fluorescence quenching agent containing DAPI was added dropwise to prevent fluorescence quenching and to stain nuclei. Images were obtained using a confocal microscope (Zeiss-LSM-980, Germany).

1.7 Hematoxylin and eosin staining

Frozen tissue sections were prepared at 10-μm thickness using a cryostat, equilibrated to room temperature, fixed with 4% neutral paraformaldehyde for 20–30 min, washed with running water for 30 s, stained dropwise with hematoxylin for 12–15 min and washed again with running water. The sections were differentiated in 1% hydrochloric acid ethanol for 5 s and soaked in tap water for 10 min, followed by addition of eosin stain for 30–40 s and washing with running water. Subsequently, gradient de-coloration was conducted by soaking in 75%, 85%, 95% and absolute ethanol for 2–5 s each. Tissue sections were made transparent by immersion in xylene for 1–2 s, dried and sealed under coverslips with 1 to 2 drops of neutral resin. Cytoplasm, collagen fibres and muscle fibres were distinguished by different shades of red, while nuclei were distinguished by blue staining.

1.8 RT-PCR

Total RNA was isolated from Sca-1+ cells treated as indicated using Nucleozol (Macherey-Nagel, Duren, Germany) according to the manufacturer's instructions. After RNA quality detection and concentration determination using a spectrophotometer, total RNA (1 µg) was reverse-transcribed into cDNA using the PrimerScriptTM RT reagent Kit with gDNA Eraser (TaKaRa). Standard PCR was used to detect the receptor subtypes expressed on Sca-1+ cells, while real-time fluorescent quantitative PCR (RT-qPCR) was used to detect changes in receptor expression induced by ATP. The specific primer pairs used for estimation of gene expression levels are presented in *Supplementary Table 1*.

1.9 Western blotting

Total membrane and phosphorylated proteins were extracted from Sca-1+ cells, and quantified using the BCA method. Samples (30 µg) were separated by sodium dodecyl sulphate polyacrylamide gel electrophoresis (SDS-PAGE) and transferred to nitrocellulose (NC) membranes. Membranes were incubated with the indicated primary antibody overnight at 4°C and then with horseradish peroxidase (HRP)-conjugated secondary antibody (1: 2000). Antibodies included anti-P2Y2R (extracellular) (1:200, Alomone), anti-P2Y6R (extracellular) (1:400, Alomone), anti-caveolin-1 (D46G3 XP®Rabbit mAb, 1:1000, Cell Signalling Technology), phospho-p44/42MAPK(Erk1/2) (Thr202/Tyr204) (D13.14.4E) XP®RabbitmAb#4370 (1:2000, Cell Signalling Technology) and p44/42 MAPK (Erk1/2) (137F5) rabbit mAb (1:1000, Cell Signalling Technology). Target protein bands were visualised using ECL chemiluminescence substrate and a gel imaging system. Expression levels were quantified relative to Caveolin or GADPH as the gel loading control.

1.10 Transcriptomics

The effect of ATP on gene expression was examined. Sca-1+ cells were cultured in 6-well dishes (10 cm) and evenly divided into control (C) and ATP (A) treatment groups. Group C was cultured in serum-free medium and group A in serum-free medium containing 30 µM ATP for 18 h. Each culture was quickly washed with PBS, de-plated by trypsinisation for 2 min (stopped with 2 mL PBS), harvested and centrifuged (5 min/137g). The supernatant was removed and 1 mL PBS was added to the precipitated cells. Cells were uniformly dispersed and transferred to 1.5 mL EP tubes for centrifugation (5 min/137g). The supernatant was again discarded and pelleted cells frozen in liquid nitrogen for 4–5 seconds, transferred to a liquid nitrogen tank and shipped on dry ice (−80ºC) to Beijing Nuozhiguan Technology for mRNA-seq analysis using the Illumina sequencing platform. After filtering the original data, checking the sequencing error rate and checking the GC content distribution, we obtained clean reads for subsequent analysis of differentially expressed genes (DEGs). No less than 6.7 Gb of clean data were obtained from each sample, and the R2 between biological replicates was greater than 0.973 in all cases. The DEGs between groups were screened using DESeq2 according to criteria

|log2(FoldChange)| > 1.0 and adjusted P (Padj) < 0.05. The threshold for functional enrichment was also Padj < 0.05. The results are publicly available at BioProject (https://www.ncbi.nlm.nih.gov/bioproject/) under accession number PRJNA900447.

2. **Table**

Table 1 Specific primer pairs for purinergic P2 receptor genes for (R-PCR) and RT-PCR reagents

Gene name	Primer Sequence
P2rx1	F:5'- CGGATGGTGCTGGTACGAAA-3'<br>R:5'- CACTGACACACTGCTGATAAGG-3'
P2rx2	F:5'- GCGTTCTGGGACTACGAGAC-3'<br>R:5'- CGTACCACACGAAGTAAAGCA-3'
P2rx3	F:5'- TTGGGATCATCAACCGAGCC-3'<br>R:5'-TGATGACAAAGACAGAGGTGCC-3'
P2rx4	F:5'- ACCAGGAAACGGACTCTGTG-3'<br>R:5'-TCACGGTGACGATCATGTTGG-3'
P2rx5	F:5'- ACCAACCTGATCGTGACTCCT-3'<br>R:5'- ACAGTCGGTGTCCTCTGAACA-3'
P2rx6	F:5'- GTAGTCTACGTGATAGGGTGGG-3'<br>R:5'- CACGAGGAAGTTGGTTACCAG-3'
P2rx7	F:5'-AGTTGGTGCCAGTGTGGAAA-3'<br>R:5'- TAGCACCTGTAAGCACGGTG-3'
P2ry1	F:5'- CCAATGTGCCCTGACCAAGA-3'<br>R:5'-ACATCCAGATAGCCACGCTG-3'
P2ry2	F:5'-GTGCTCTACTTCGTCACCA-3'<br>R:5'-GACCTCCTGTGGTCCCATAA-3'
P2ry4	F:5'- AGACGGGCCTGATGTGTATC-3'<br>R:5'- AGGTTCACATGCCCTGTACC-3'
P2ry6	F:5'- GGGTAGTGTGTGGAGTCGTG-3'<br>R:5'-GCGAGTAGACAGGATGGGTG-3'
P2ry10	F:5'- GCGTAGGTACGATGTGGGC-3'<br>R:5'-GCAAGAATCTGTGCTATTGGCT-3'

3. **Supplemental Figures:**

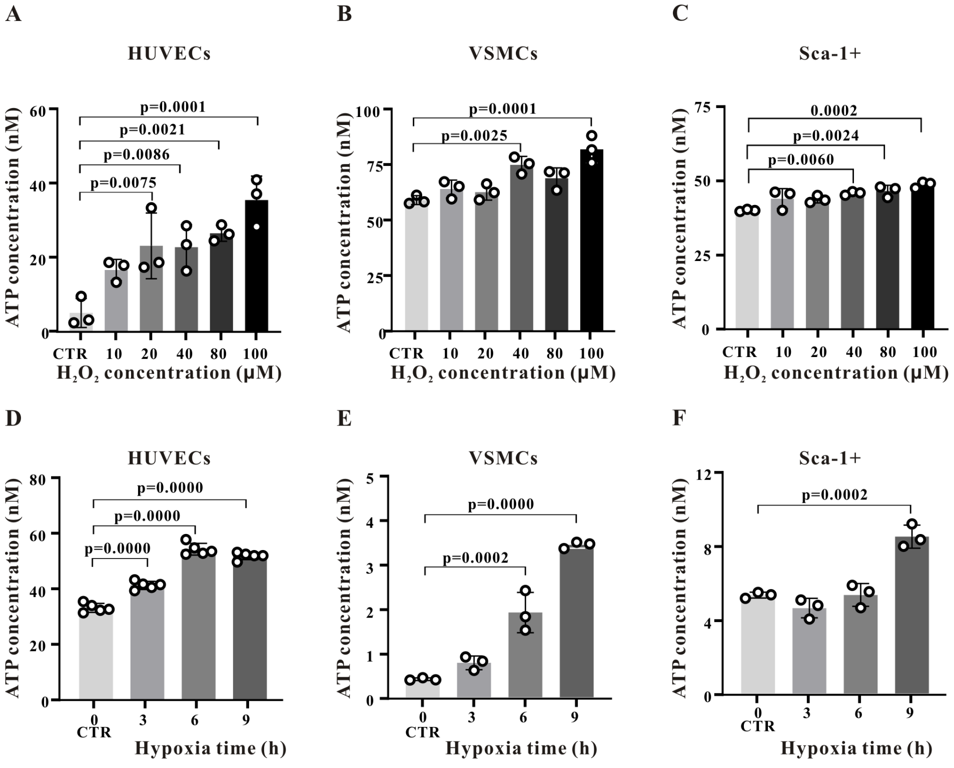

Fig. S1 **ATP release from Sca-1+ cells subjected to oxidative stress and hypoxia. A, B and C, Accumulation of extracellular ATP in cultures of human umbilical vein endothelial cells (HUVECs), vascular smooth muscle cells (VSMCs) and Sca-1+ stem cells over 12 h during treatment with different concentrations of hydrogen peroxide (H2O2). D, E and F, Accumulation of extracellular ATP after 3, 6 and 9 h of hypoxia. Values are mean ± SD of 3–5 biological replicates. Summary data were obtained from 3-4 independent experiments and data were first tested by Shapiro-Wilk test for normality and Ordinary one-way ANOVA (Dunnett's multiple comparisons test) was performed. A P< 0.05 is considered a statistically significant test, and statistically significant P-values between the two groups are shown on the graph.**

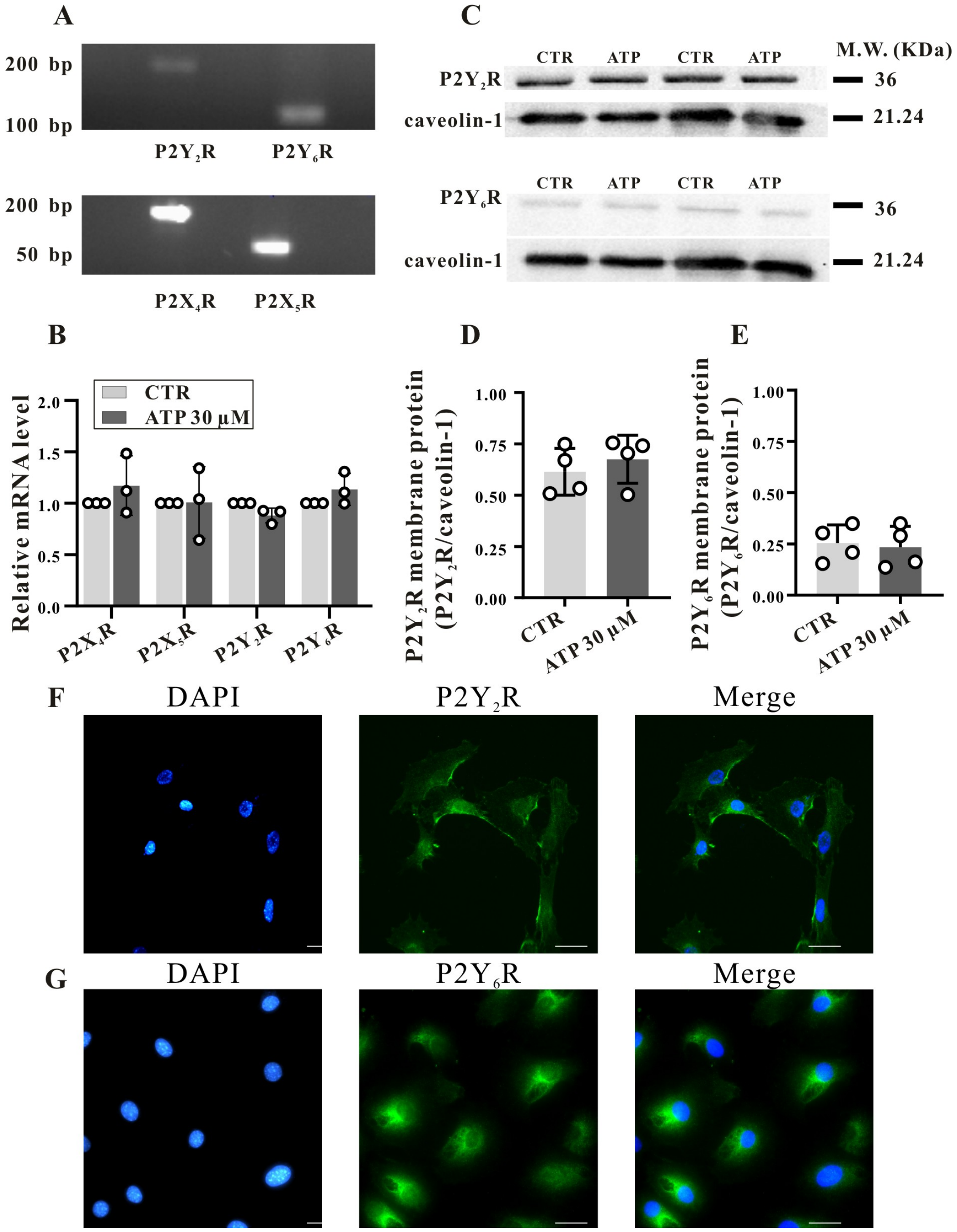

Fig. S2 **mRNA and protein expression of P2R subtypes on Sca-1+ cell membranes. A. Agarose gel electrophoresis of P2XRs and P2YRs in the membrane fraction of Sca-1+ cell lysate. B. Summary of P2X4R, P2X5R, P2Y2R and P2Y6R mRNA expression levels by Sca-1+ cells, and the influence of ATP treatment on expression as measured by real-time quantitative PCR. P-values in B were calculated using the Mann-Whitney test and the effects of each group were compared to the respective control (n = 3 independent experiments). C, Western blot showing membrane P2Y2R and P2Y6R protein expression by Sca-1+ cells and changes in expression after ATP treatment. D and E, Histograms summarizing Western blotting results (n = 4 independent experiments, respectivly). P-values were calculated using the unpaired test. F and G, Immunofluorescence images of P2Y2R and P2Y6R on Sca-1+ cells. P2Y2R and P2Y6R were labelled by green fluorescence, and the cell nucleus was labelled with blue DAPI fluorescence (400×).**

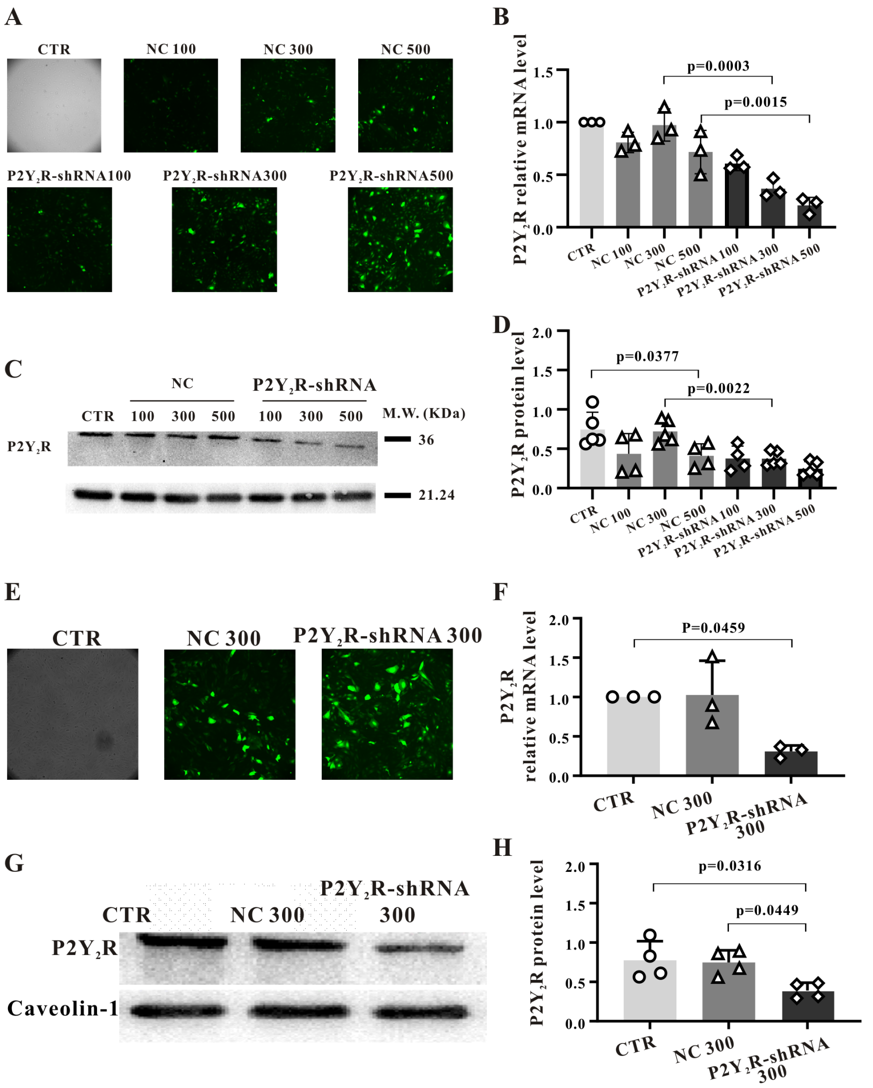

Fig. S3 **Testing for the optimal P2Y2R-shRNA transfection dose. A, Sca-1+ cells transfected with P2Y2R-shRNA or the control shRNA emitted green fluorescence after 48 hours (×100); B, Quantitative RT-PCR showing P2Y2R mRNA knockout efficiency after 48 hours. P-values were calculated using the Mann-Whitney test and the effects of each group were compared to the respective control (n = 3 independent experiments). C and D, Western blot showing protein knockout efficiency after 72 hours with MOIs of 100, 300 and 500. Data in D were first tested by Shapiro-Wilk test for normality and then Ordinary one-way ANOVA (Tukey's multiple comparisons test) was performed (n = 5 independent experiments). E, F, G and H, Effect of 300 MOI P2Y2R-shRNA on P2Y2R mRNA and protein expression by Sca-1+ cells. Sca-1+ cells transfected with P2Y2R-shRNA and NC showed green fluorescence (E, ×100). Quantitative RT-PCR showing the mRNA knockout efficiency in Sca-1+ cells transfected with P2Y2R-shRNA adenovirus for 48 hours at MOI = 300. (F). P-values in F were calculated using the Nonparametric test (Kruskal-Wallis test) (n = 3 independent experiments). Western blot showing the effect of P2Y2R-shRNA adenovirus at MOI = 300 on P2Y2R protein expression by Sca-1+ cells at 72 hours (G and H). Data in H were first tested by Shapiro-Wilk test for normality and then Ordinary one-way ANOVA (Tukey's multiple comparisons test) was performed. A P< 0.05 is considered a statistically significant test, and statistically significant P-values between the two groups are shown on the graph. (n = 3 independent experiments).**

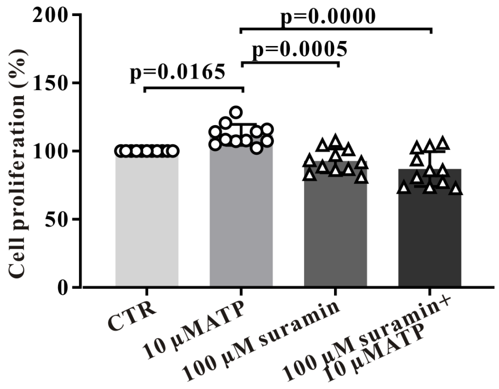

Fig. S4 **Reduction in ATP-induced Sca-1+ cell proliferation by non-specific P2R blockade. Suramin was added 5 min prior to ATP. Values shown are mean ± SD relative to untreated controls (n = 11 independent experiments). P-values were calculated using the Nonparametric test (Kruskal-Wallis test).**

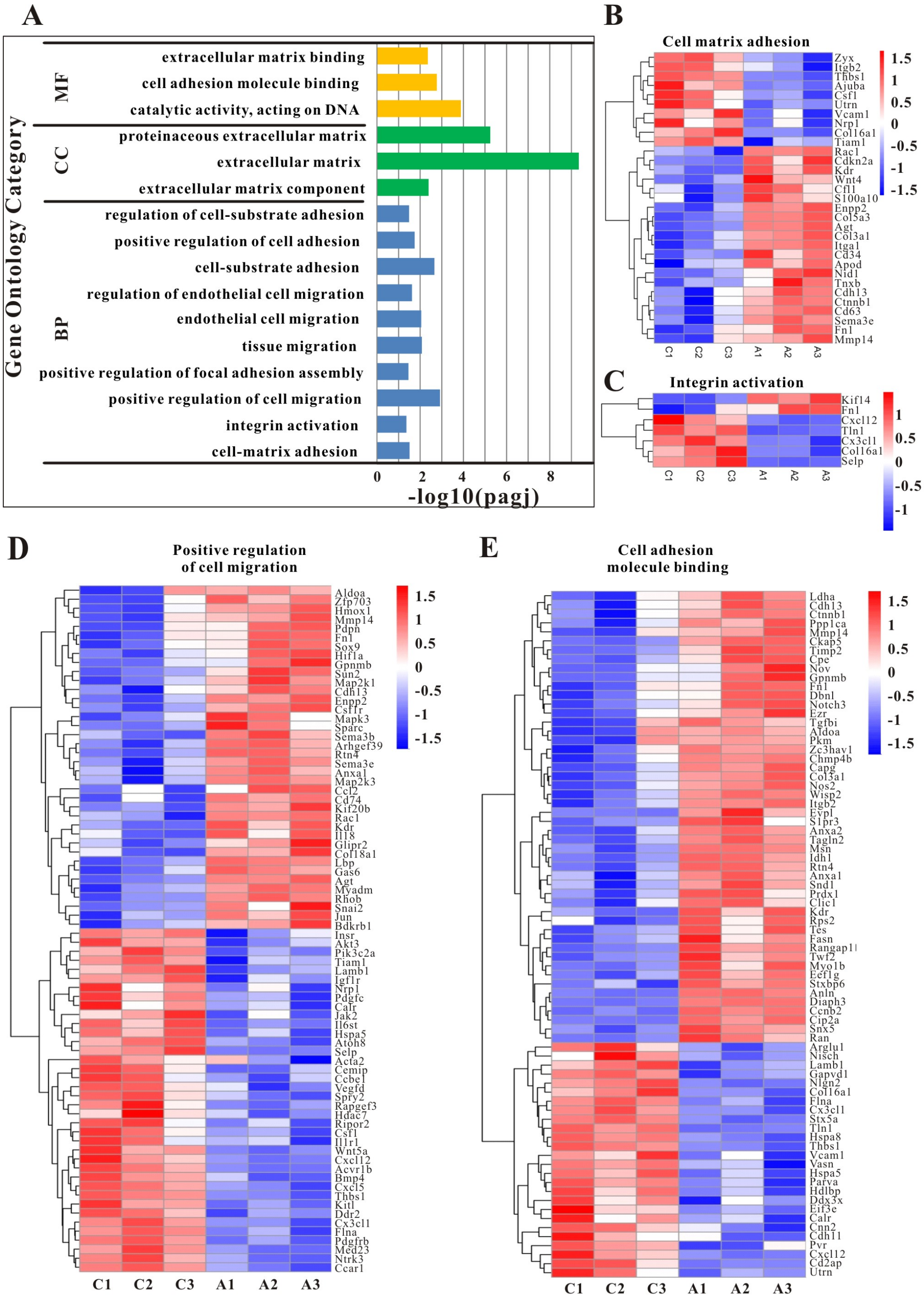

Fig. S5 **Gene Ontology (GO) terms related to cell migration or proliferation for genes differentially expressed between ATP-treated and control Sca-1+ cells. A, ATP altered the expression levels of genes in the main functional pathways controlling Sca-1+ cell migration. The y-axis is the GO classification and the x-axis is the significance level of GO classification enrichment expressed as -log10 (Padj). Different colours represent BP, CC and MF. B, Genes upregulated or downregulated by ATP associated with cell matrix adhesion. C, Genes upregulated or downregulated by ATP associated with integrin activation. D, Genes upregulated or downregulated by ATP implicated in the positive regulation of cell migration. E, Genes upregulated or downregulated by ATP associated with cell adhesion molecule binding. C1, C2 and C3 represent the three biological replicates of the control group, and A1, A2, A3 represent the three biological replicates of the ATP-treated group.**

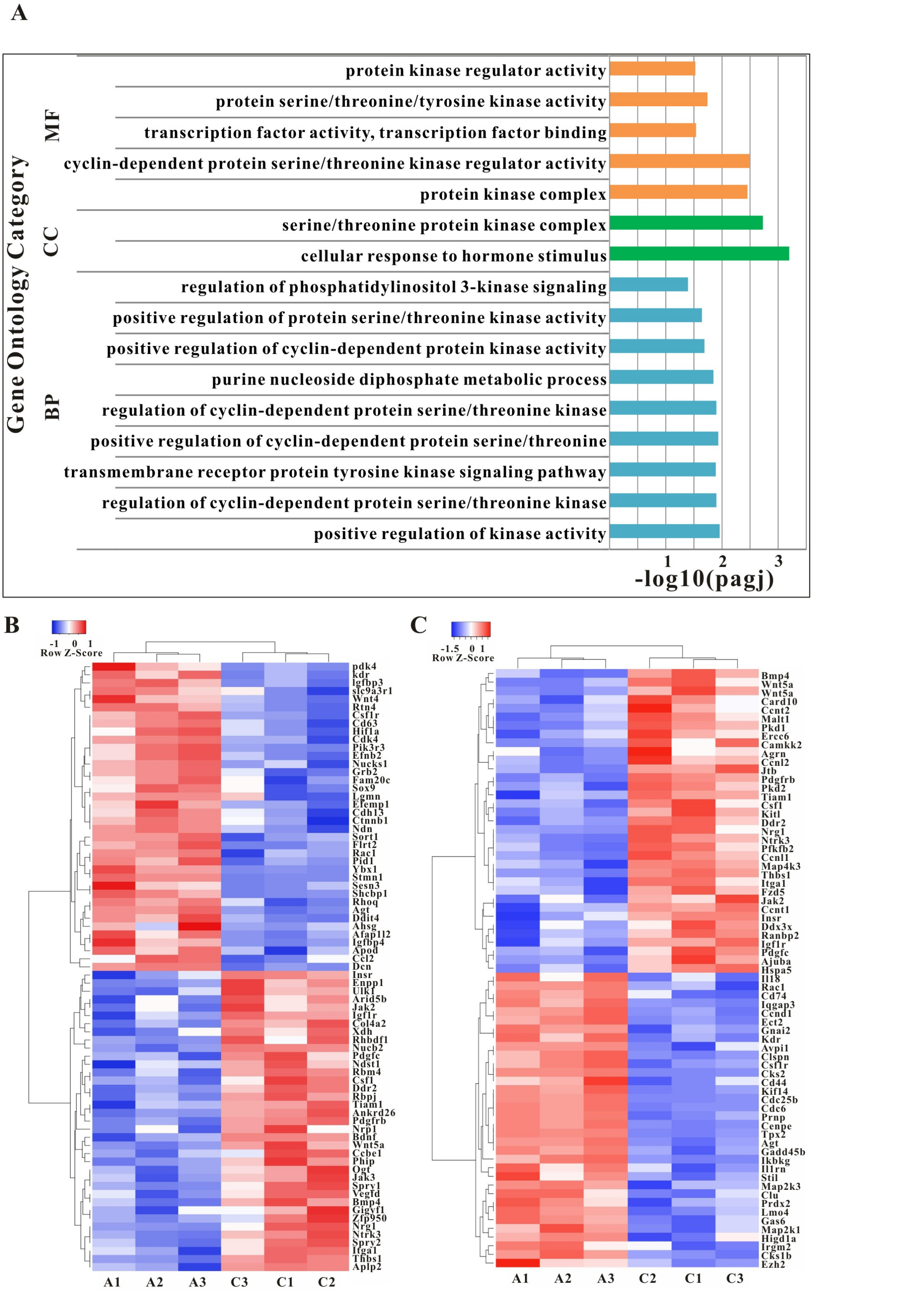

Fig. S6 **Gene Ontology (GO) terms related to cell kinase activity for genes differentially expressed between ATP-treated and control Sca-1+ cells. A, Major functional pathways involved in cell cycle-related protein kinase activity. B, Genes upregulated or downregulated by ATP in the transmembrane receptor protein tyrosine kinase signalling pathway. C, Genes upregulated or downregulated by ATP implicated in the positive regulation of kinase activity. C1, C2 and C3 represent the three biological replicates of the control group, and A1, A2 and A3 represent the three biological replicates of the ATP treatment group.**

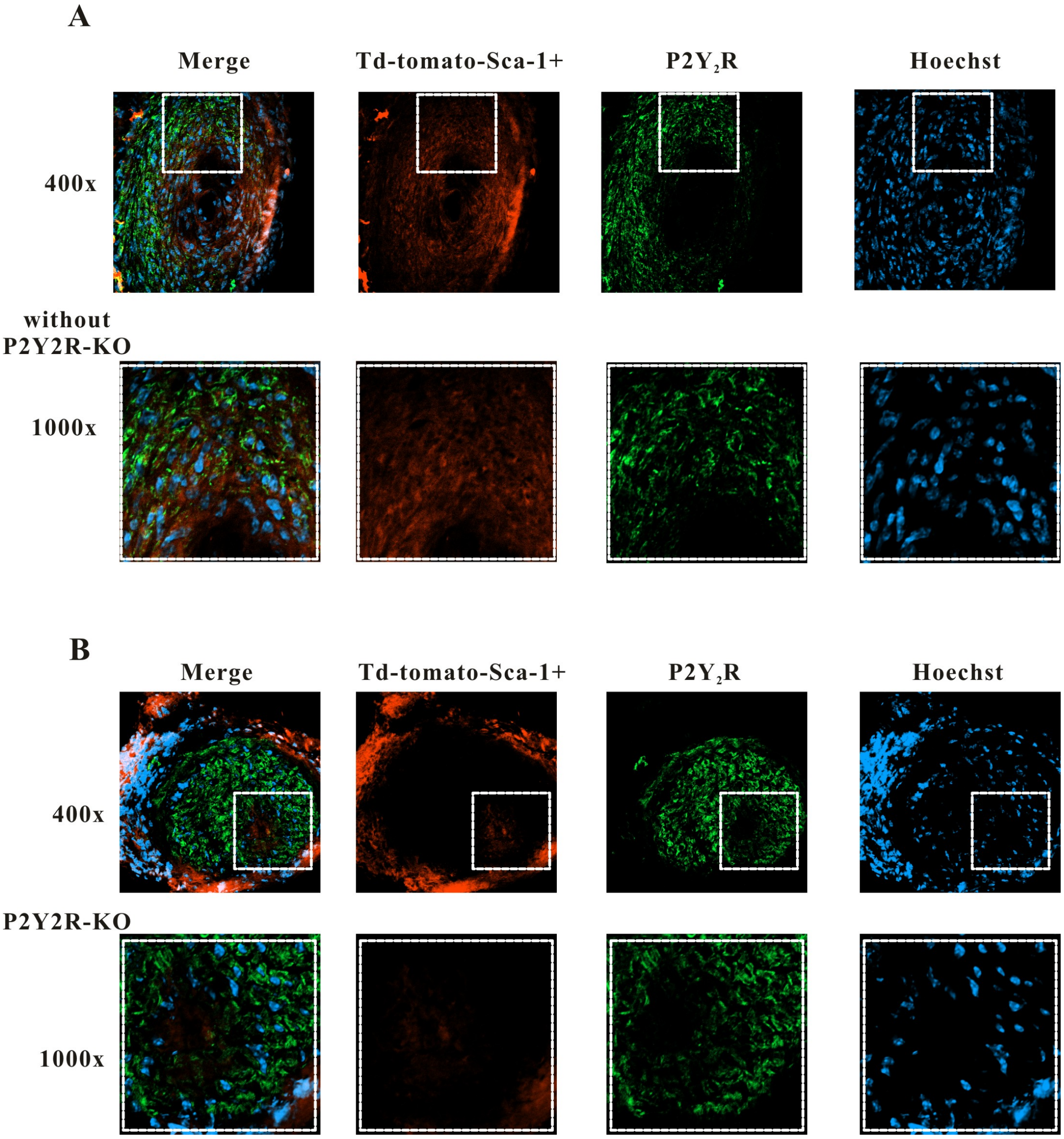

Fig. S7 **Confirmation of P2Y2R knockdown in mouse femoral artery. A, Immunofluorescence images of a femoral artery section from a Sca-1+-Cre × Rosa26-TdTomato mouse at 3 weeks post-injury, B, Immunofluorescence images of a femoral artery section from a P2Y2R-flox×Sca-1-CreERT×TdTomato mouse at 3 weeks post-injury showing tamoxifen-induced P2Y2R knockout in Sca-1+ cells. Red, TdTomato (Sca-1+ cell); Green, P2Y2R; Blue, Hoechst 33342.**

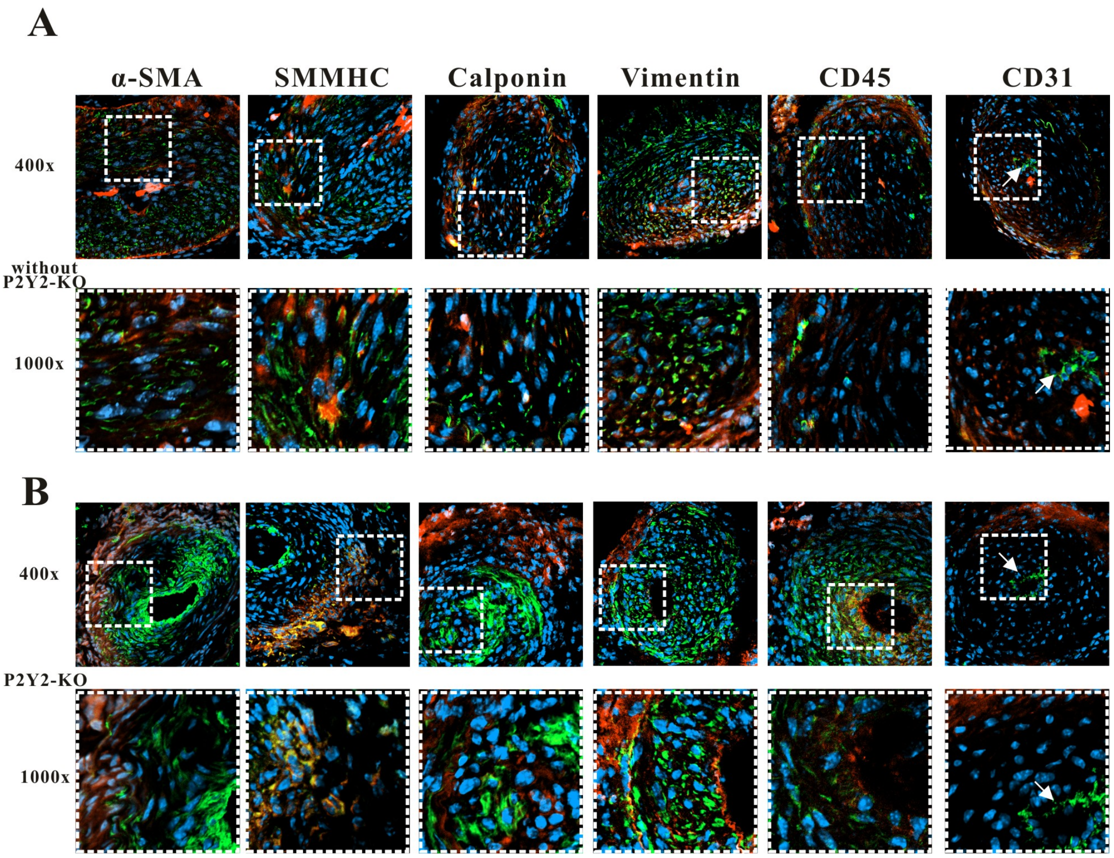

Fig. S8 **Sca-1+ cell differentiation into smooth muscle cells, inflammatory cells, fibroblasts and endothelia cells. Red, TdTomato (Sca-1+ cell); Blue, Hoechst 33342; Green, corresponding cell-type markers indicated in the figures. Smooth muscle cells marker is represented by α-SMA, SMMHC or Calponin; fibroblasts marker is represented by Vimentin; inflammatory cells marker is represented by CD45; endothelia cells marker is represented by CD31. Merger of red and green (yellow) indicates that the indicated marker is expressed in Sca-1+ cells.**
